# Microglia maintain the normal structure and function of the hippocampal astrocyte network

**DOI:** 10.1002/glia.24179

**Published:** 2022-04-08

**Authors:** Yixing Du, Faith H. Brennan, Phillip G. Popovich, Min Zhou

**Affiliations:** ^1^ Department of Neuroscience The Ohio State University Wexner Medical Center Columbus Ohio USA; ^2^ Center for Brain and Spinal Cord Repair The Ohio State University Wexner Medical Center Columbus Ohio USA

**Keywords:** astrocytes, hippocampus, microglia, synaptic plasticity, synaptic transmission

## Abstract

Microglial control of activity‐dependent plasticity and synaptic remodeling in neuronal networks has been the subject of intense research in the past several years. Although microglia–neuron interactions have been extensively studied, less is known about how microglia influence astrocyte‐dependent control over neuronal structure and function. Here, we explored a role for microglia in regulating the structure and function of the astrocyte syncytium in mouse hippocampus. After depleting microglia using a CSF1R antagonist (PLX5622, Plexxikon), we observed severe disruption of astrocyte syncytial isopotentiality and dye coupling. A decrease in astrocyte‐specific gap junction connexin (Cx) 30 and 43 expression, at least partially accounts for these microglia‐dependent changes in astrocytes. Because neuronal function requires intact astrocyte coupling, we also evaluated the effects of microglia depletion on synaptic transmission in the hippocampus. Without microglia, the strength of synaptic transmission was reduced at baseline and after long‐term potentiation (LTP). Conversely, priming microglia with systemic injections of lipopolysaccharide enhanced CA3‐CA1 synaptic transmission. This microglia‐induced scaling of synaptic transmission was associated with increased expression of post‐synaptic scaffold proteins (Homer1) in CA1. However, astrocyte network function was not affected by microglia priming, indicating that microglia‐dependent effects on astrocytes and neurons vary across functional states. Through manipulation of microglia in the brain, our results reveal the importance of microglia in homeostatic regulation of the astrocyte syncytium and scaling of synaptic transmission. These novel mechanisms uncover a new direction for future studies interrogating microglia function in various physiological and pathological contexts.

## Main Points

- Microglia maintain expression of astrocyte connexins, gap junctional coupling, and astrocyte syncytial isopotentiality in mouse hippocampus.
- Microglia enable bidirectional scaling of synaptic transmission, partly by modulating postsynaptic Homer1.

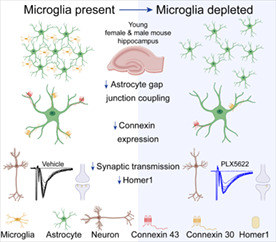

## 1 INTRODUCTION

Microglia regulate activity‐dependent plasticity and synaptic remodeling of neuronal networks in the brain during development and in adults in both health and disease. Specifically, in response to neuronal cues (e.g., fractalkine and ATP), microglia monitor the health and function of neurons and regulate synaptic strength and connectivity of neural networks (Badimon et al., 2020; Basilico et al., 2022; Cserep et al., 2020; Eyo et al., 2014; Liu et al., 2021; Wu et al., 2020). These microglia–neuron interactions have been extensively studied in hippocampus, although functionally important microglia‐dependent effects have also been documented in spinal cord, motor cortex and visual cortex (Chen et al., 2014; Clark et al., 2015; Liu et al., 2021; Zhou et al., 2019).

Microglia can also modify neuronal structure and activity indirectly via crosstalk with astrocytes (Akiyoshi et al., 2018; Chung et al., 2013; Eroglu & Barres, 2010; Liddelow et al., 2017; Pascual et al., 2012; Rothhammer et al., 2018; Vainchtein et al., 2018). Under basal conditions, astrocytes uptake and recycle neurotransmitters and metabolites, and maintain ion homeostasis, to control neuronal excitability and synaptic transmission (Mayorquin et al., 2018; Steinhauser et al., 2012; Szabo et al., 2017). The cytoplasm of adjacent astrocytes is coupled through gap junction channels, which permit the redistribution of elevated K^+^ from sites of excessive neuronal activity to sites of lower extracellular K^+^ concentration (Ma et al., 2016; Steinhauser et al., 2012). This continuous astrocyte network, or “syncytium”, constantly equalizes the membrane potential of individual astrocytes to maintain a steady driving force for K^+^ and neurotransmitter clearance, a property known as “syncytial isopotentiality” (Huang et al., 2018; Kiyoshi et al., 2018; Ma et al., 2016). Syncytial isopotentiality is a prerequisite for optimal crosstalk between neurons and astrocytes, and the coupled astrocyte network is an effective neuroprotective mechanism for preventing or delaying spreading depolarizations (Bellot‐Saez et al., 2017; Huguet et al., 2016; Martinez‐Banaclocha, 2020). However, whether microglia support the connectivity or functionality of the astrocyte network is unknown.

Here, we aimed to understand the influence of microglia on basal expression of astrocyte connexins, gap junctional coupling, and astrocyte syncytial isopotentiality in mouse hippocampus. Since the astrocyte network strengthens synaptic plasticity (Kiyoshi & Zhou, 2019), we also investigated the effects of microglia on synaptic transmission and plasticity, both at rest (homeostasis) and in the context of inflammation. To this end, we used pharmacological microglia depletion and LPS‐induced microglial priming to explore the role of microglia in regulating electrophysiology in the young adult mouse hippocampus in situ. We performed patch‐clamp recording of individual hippocampal astrocytes, and field potential recordings of glutamatergic projection from CA3 pyramidal cells onto CA1 stratum radiatum (Schaffer collaterals), an integral pathway for memory formation in the brain (Deng et al., 2010).

Our data reveal that pharmacological ablation of microglia using PLX5622, an orally bioavailable CSF1R antagonist, weakens astrocyte syncytial isopotentiality and gap junctional coupling. This phenotype is associated with reduced strength of synaptic transmission in whole‐field recordings after LTP induction and downscaled synaptic transmission, that is, a coordinated decrease in all synaptic inputs onto a neuron. Conversely, priming microglia with systemic lipopolysaccharide (LPS) strengthens synaptic transmission at baseline and after LTP induction and upscales synaptic transmission. Recording and immunohistochemical data show that microglia control connexin expression, glutamatergic synapse density, and expression of post‐synaptic scaffold proteins in CA1. Together, these data reveal a novel role for microglia in regulating intercellular communication and neurotransmission within the hippocampus. Future work exploring and manipulating mechanisms of microglia‐dependent astrocyte syncytial isopotentiality and synaptic scaling could promote neuroprotection, plasticity, or repair in diverse neurological diseases.

## 2 METHODS

### 2.1 Mice and microglia manipulations

#### 2.1.1 Mice

Experimental procedures were performed in accordance with The Ohio State University Institutional Animal Care and Use Committee and the ARRIVE guidelines. Male and female mice at postnatal day (P) 25–45 were used at the time of recording or anatomical analysis. Mice were group housed under conventional conditions on a 12 h light–dark cycle with free access to food and water.

#### 2.1.2 Microglia depletion

**FIGURE 1:**
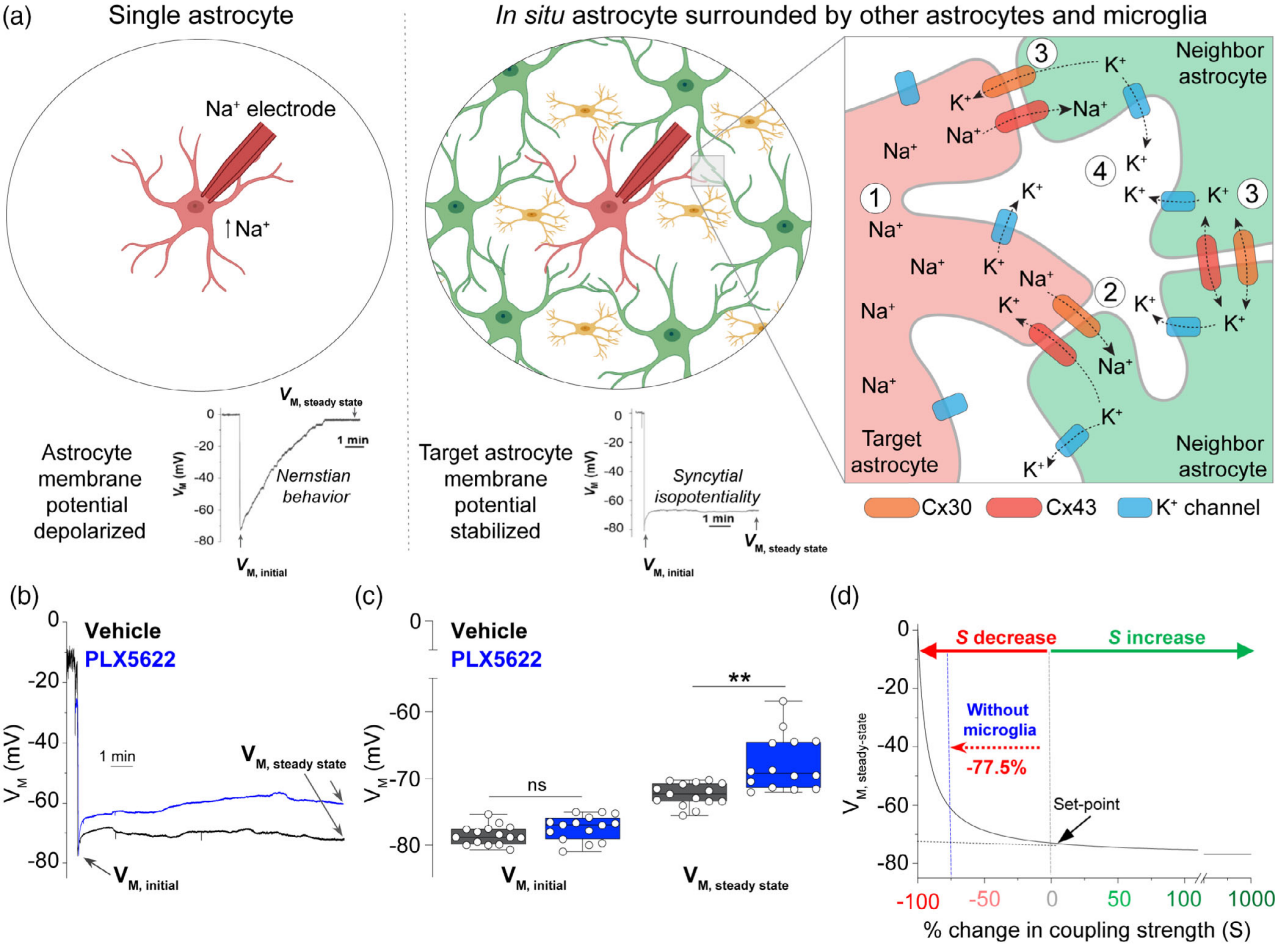
Microglia ablation weakens astrocyte gap junctional coupling. (a) Schematic showing that a Na^+^ electrode causes membrane potential depolarization (Nernstian behavior) of an isolated astrocyte, but the membrane potential remains stable (syncytial isopotentiality) when the target astrocyte is in situ and surrounded by other astrocytes and microglia. The inset shows the mechanism for syncytial isopotentiality: (1) Na^+^ loading depolarizes the target astrocyte membrane potential; (2) neighboring astrocytes repolarize the target astrocyte membrane potential through gap junctional coupling (e.g., Connexin (cx)30 and Cx43); (3) ions redistribute throughout the astrocyte network via gap junctions; (4) K^+^ ions passively move extracellularly through K^+^ channels. (b) Representative traces of astrocyte *V*_M_ recorded with K^+^ free‐Na^+^ containing electrode [Na^+^]_P_ from mice in vehicle (black trace) or PLX5622 (blue trace) groups. The initial *V*_M_ (*V*_M, initial_) was determined immediately after the breakthrough of the cell membrane in whole‐cell recording. The steady‐state *V*_M_ (*V*_M, steady‐state_) was determined 10–15 min after the membrane breakthrough and used as a readout of gap junctional coupling strength between astrocytes. (c) Microglia ablation did not alter the *V*_M, initial_, or astrocyte resting *V*_M_, but significantly shifted the *V*_M, steady‐state_ toward depolarization, or the closing state of syncytial coupling. (d) Computational modeling shows that changes in coupling strength (*S*) are exponentially correlated to the deviation of *V*_M, steady‐state_ from the set‐point (−73 mV) in [Na^+^]_P_ recording (Kiyoshi et al., 2018). This model predicts a 77.5% inhibition of astrocyte syncytial coupling after microglia depletion. Two‐sided Student's t‐test or Aspin‐Welch unequal‐variance test, *n =* 14–15 recorded cells per group, **p < .01

**FIGURE 2:**
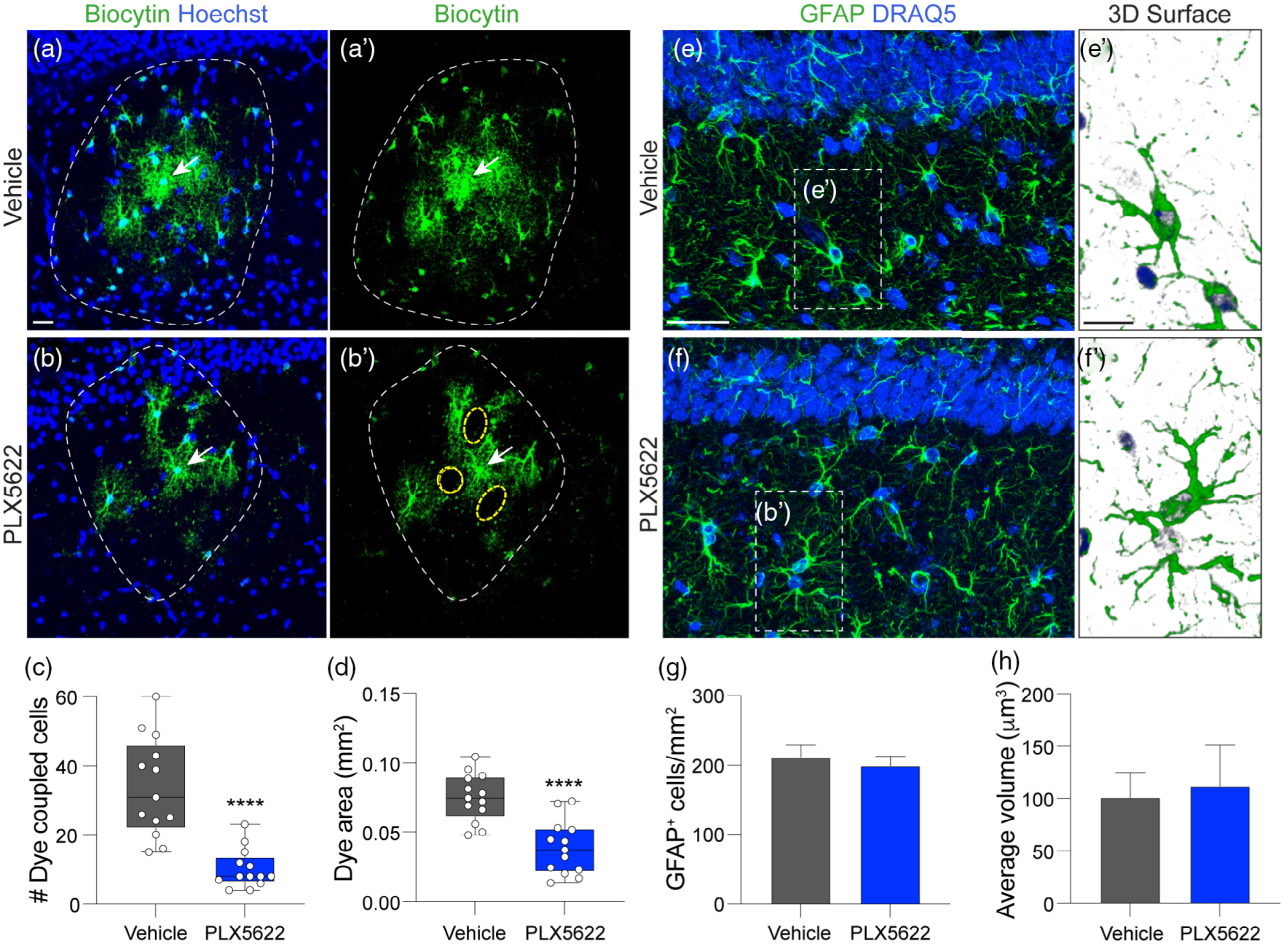
Microglia depletion impairs astrocyte coupling without altering astrocyte number or morphology. (a,b) A target astrocyte (arrow) was filled with biocytin. Coupled cells were visualized by biocytin staining (green fluorescent). The coupled cell defined areas were indicated by white dash lines; and the areas devoid of biocytin around the target astrocyte were indicated by yellow dash lines. C, D: Fewer biocytin‐Cy2^+^ cells (c) and reduced dye‐labeled area was observed after microglia ablation (d). (c,d) Student's two‐sided *t*‐test or Aspin‐Welch unequal‐variance test, *n =* 13 slices from 4 mice/group, ****p < .0001. (e,f) Representative high magnification confocal images of GFAP staining in the stratum radiatum of mice fed vehicle (e) or PLX5622 (f). Scale bar (in e) = 25 μm; (in e') = 20 μm. (e', f') Three‐dimensional reconstructions of astrocytes were generated to determine cell volume. Green = GFAP, semi‐transparent blue = DRAQ5^+^ nuclei. (g, h) Quantification revealed there was no difference in the number (g) or cell volume (h) of GFAP^+^ cells with astrocyte morphology in the hippocampus. (g,h) Student's two‐sided *t*‐test, *n =* 4 mice/group

**FIGURE 3:**
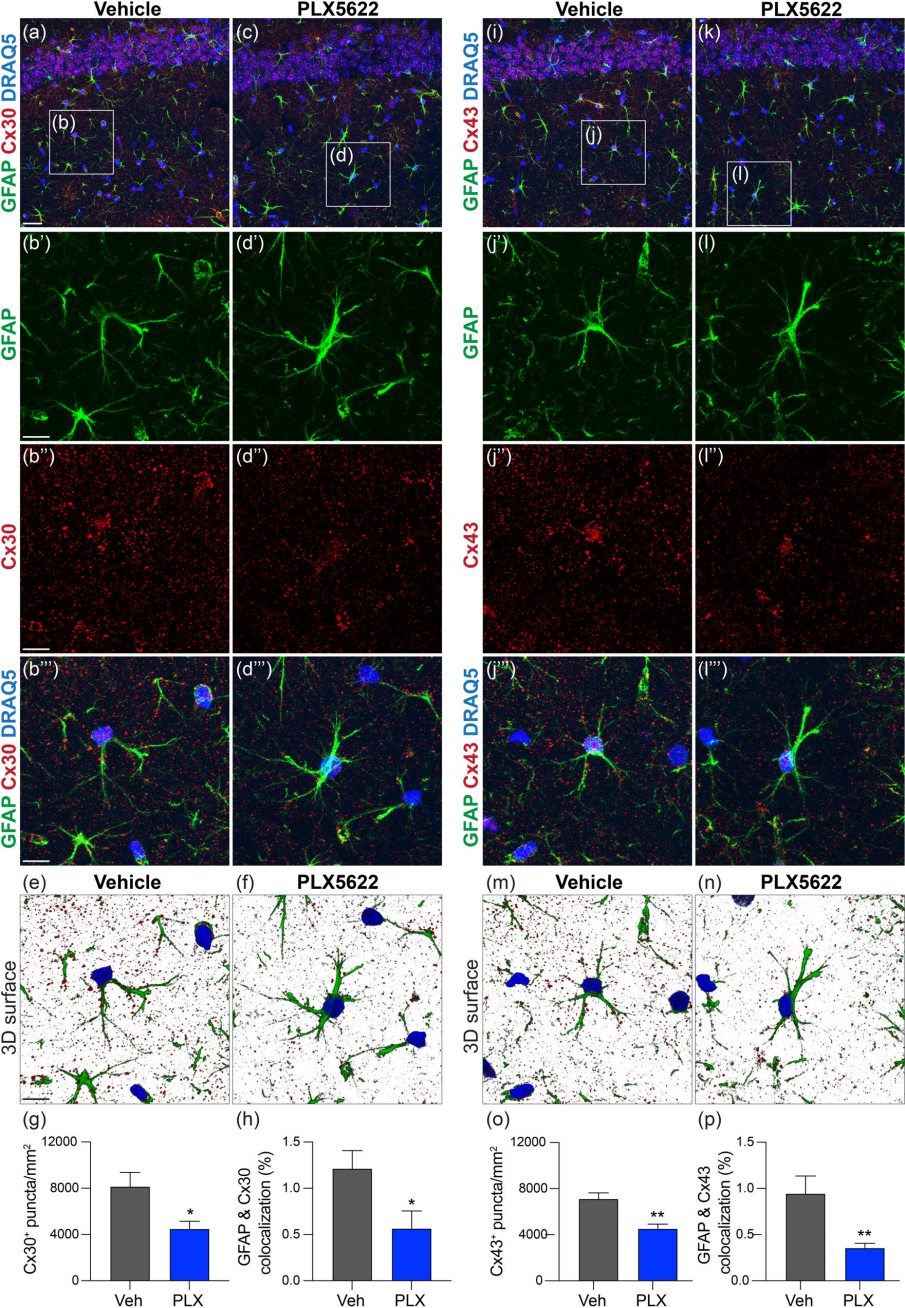
Microglia depletion reduces hippocampal and astrocyte connexin expression. (a–d) Confocal images of connexin 30 (Cx30) expression in the hippocampus in mice fed vehicle (a, b) or PLX5622 (c, d). Scale bar a, c, i, k (in a): 25 μm. Area quantification (g) and colocalization analysis (e, f, h) show microglia depletion reduces Cx30 expression in the hippocampus (g) and specifically on astrocytes (h). (i‐p) Confocal images of connexin 43 (Cx43) expression in the hippocampus in mice fed vehicle (i, j) or PLX5622 (j, k). Area quantification (o) and colocalization analysis (m, n, p) show microglia depletion reduces Cx43 expression in the hippocampus (o) and specifically on astrocytes (p). Scale bar b'–f, j'–n (in b') = 10 μm. (g, h, o, p) Student's two‐sided t tests, *n =* 5 regions of interest within the CA1 stratum radiatum (g, o) and *n =* 5 astrocytes (h, p) per mouse were analyzed and averaged from *n =* 5–7 mice/group, *p < .05, **p < .01

**FIGURE 4:**
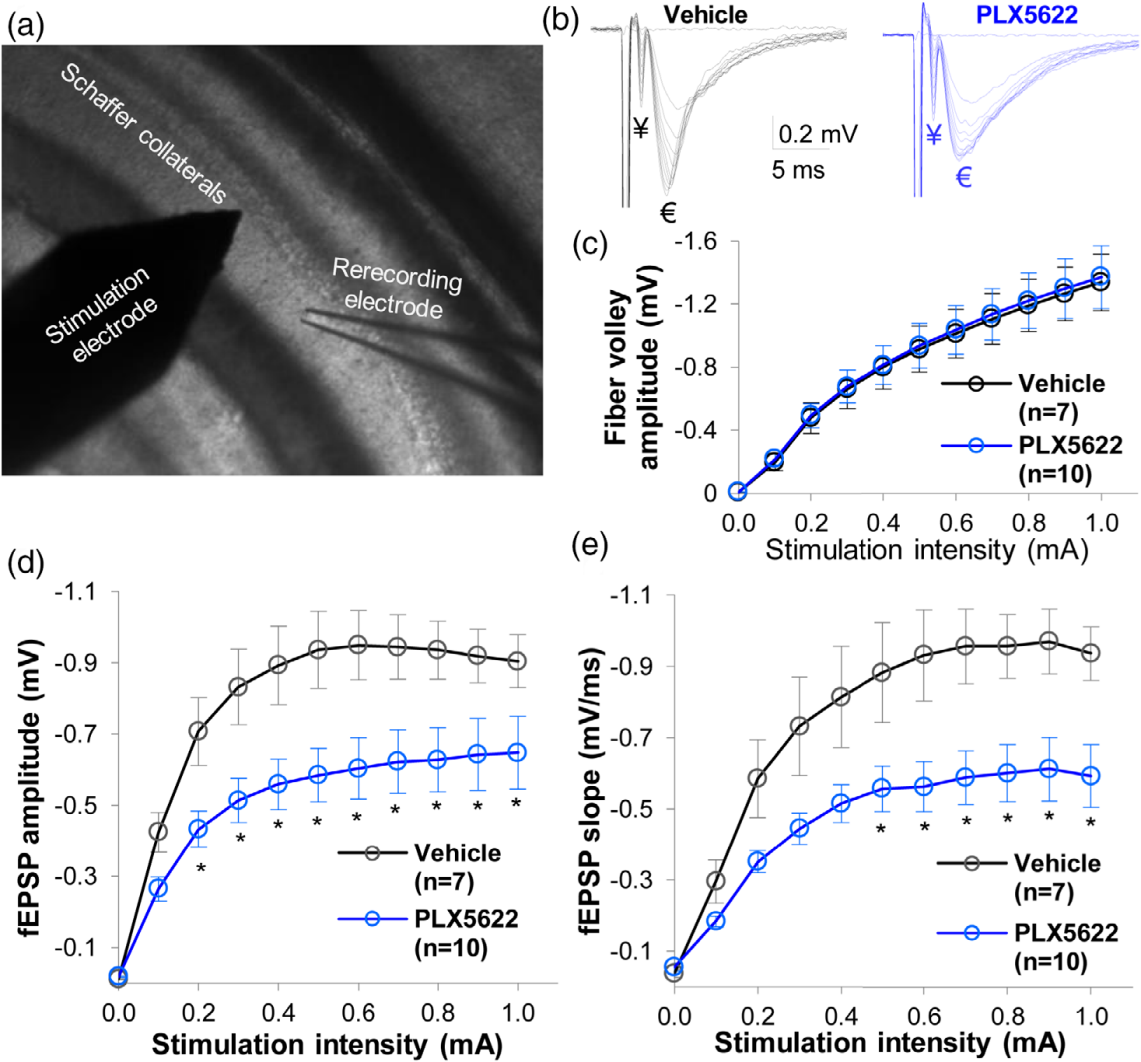
Microglia ablation weakens CA3 to CA1 synaptic transmission in hippocampus. (a) Experimental layout for recording field excitatory synaptic potentials (fEPSPs) in the hippocampus. The stimulation electrode was placed on CA3 axonal projections to the CA1 region (Schafer collaterals (SC)), and the stimulation‐elicited fEPSPs were recording in the CA1 *stratum radiatum*. (b) The fiber volley (¥) and the subsequent fEPSP (€) elicited by various stimulation intensities in vehicle (black trace) and PLX5622 (blue trace) groups. (c) The input–output relationship between stimulation intensity and presynaptic fiber volley amplitude. Input here refers to graded electrical stimulation of SC, and output refers to SC stimulation‐induced fiber volley amplitudes. Input–output curves were superimposed in vehicle and PLX5622 groups, indicating intact function of presynaptic axons after microglia depletion. (d–e) Input–output relationships between stimulation intensity and fEPSP amplitude (d) and slope (e) in the CA1 region. Input refers to graded electrical stimulation of SC, whereas output refers to SC stimulation induced fEPSPs. The fEPSP outputs show a significant decrease in mice fed PLX5622 compared to vehicle mice. Two‐way repeated measures ANOVA, *n =* 7–10 recorded hemisphere brain slices per group, *p < .05

**FIGURE 5:**
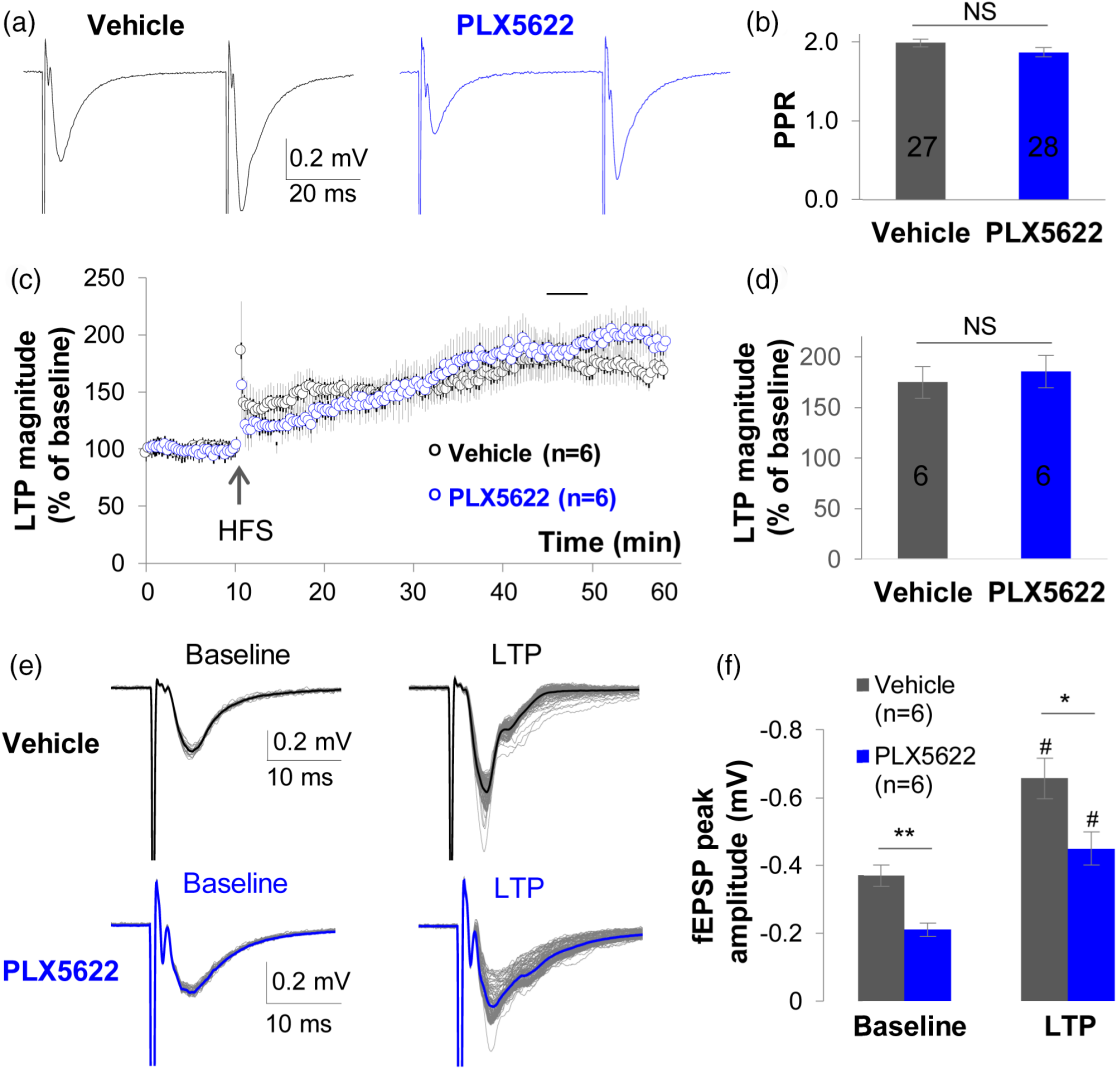
Microglia ablation alters the strength of synaptic transmission, but not synaptic plasticity. (a) Representative traces of paired‐pulse facilitation (PPF) experiments of CA3 to CA1 synaptic transmission: Two consecutive fEPSPs were induced by SC stimulation at 40 ms intervals in vehicle and PLX5622 groups. The amplitudes of the induced fEPSPs were noticeably smaller in the PLX5622 group, however, the ratio of paired‐pulse facilitation (PPR) did not differ between the two groups (b). (c) After delivery of two trains of 100 Hz high frequency stimulation (HFS, arrow), long‐term potentiation (LTP) could be induced in the CA1 region in both vehicle and PLX5622 diet groups. Prior to HFS, 5 min baselines were established from stably recorded fEPSP slope. After HFS, fEPSPs were continuously recorded for 50–60 min, and the potentiation of fEPSP slope from 36 to 40 min post HFS (a short line indicated in the time course of [c]) were analyzed and compared to baseline and between the two groups. (d) The percentage of induced LTP did not differ between vehicle and PLX5622 diet groups. E. Representative overlaid recordings of fEPSPs in the baseline, that is, 0–10 min before HFS in (c), and LTP after HFS, that is, 11–55 min in (c), in vehicle and PLX5622 diet groups. (f) In both baseline and LTP stage, microglia ablation weakens CA3 to CA1 synaptic transmission. ^#^ *P* < .05 comparing to baseline in same group, paired‐sample Student's *t*‐test; *p < .05, **p < .01 comparing between groups, two‐sided Student's *t*‐test. The numbers in the graphs indicate number of recorded brain slices in each group

A colony stimulating factor 1 receptor (CSF1R)‐ antagonist, PLX5622 (Plexxikon Inc., formulated in AIN‐76A standard chow by Research Diets Inc. at 1200 ppm) was used to pharmacologically deplete microglia. PLX5622 is an orally bioavailable, blood–brain barrier permeable compound that specifically inhibits CSF1R tyrosine kinase activity with 50‐fold selectivity over four related kinases (Dagher et al., 2015; Green & Hume, 2021). Using QuickCalcs (GraphPad software), mice were randomly assigned to cages then cages were randomly assigned to receive either Vehicle or PLX5622 diet. Immediately after weaning (p21), mice received PLX5622 diet or Vehicle diet for 7 days continuously until recording (Figures 1, 2, 3, 4, 5, 8, S1, and S2).

#### 2.1.3 Microglia priming

**FIGURE 6:**
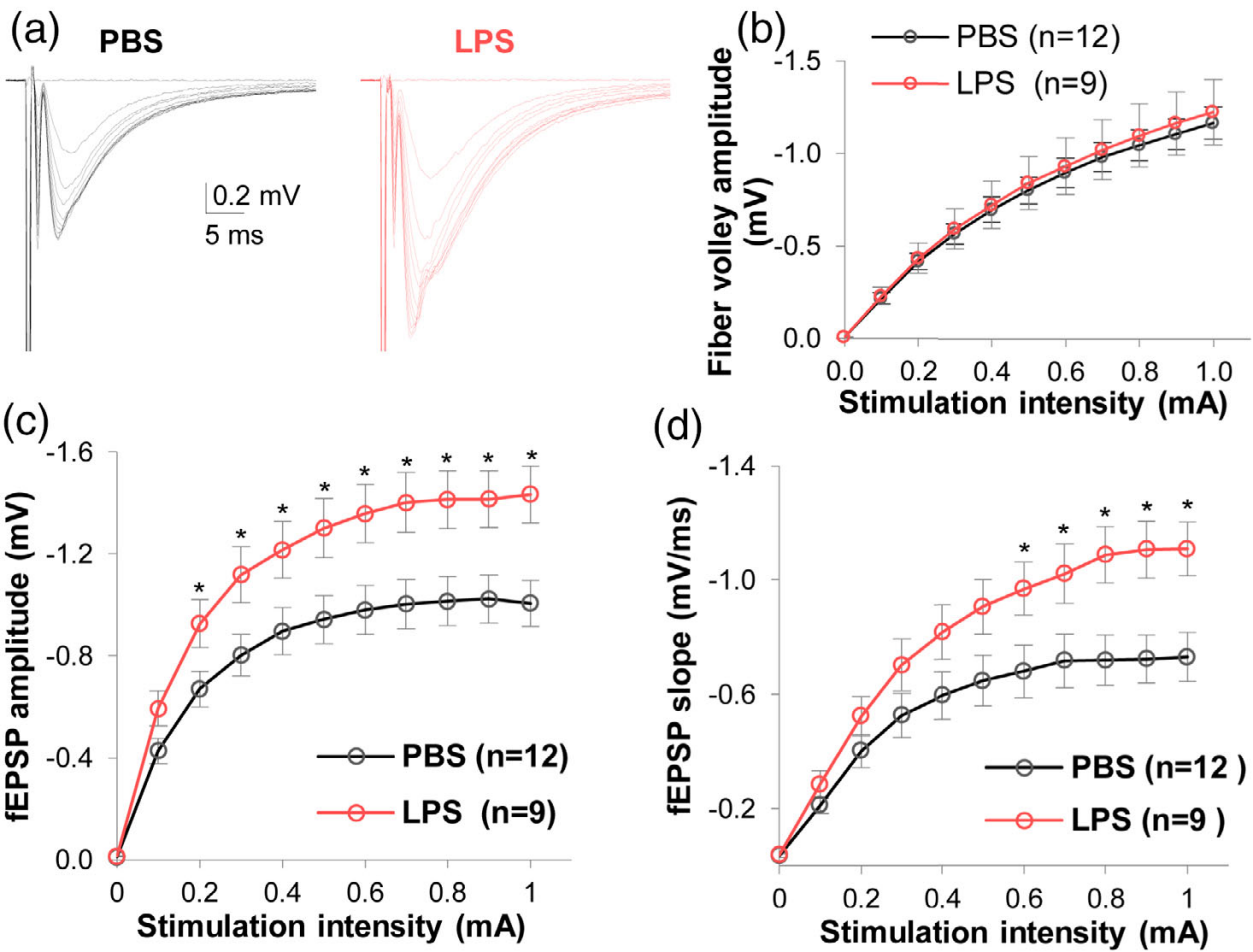
Microglia priming with LPS up‐scales synaptic transmission. (a) Representative traces showing fEPSPs elicited by stimulation of varying intensity in the CA1 region; the fEPSPs were noticeably larger in mice injected with LPS compared to PBS injected mice. (b) The relationship between stimulation‐intensity and fiber volley amplitude revealed identical response curves in PBS and LPS groups, indicating LPS does not change Schaffer collateral axonal integrity. (c,d) Input–output relationships between stimulation intensity and fEPSP amplitude (c) and slope (d) in the CA1 region. Input refers to graded electrical stimulation of SC, whereas output refers to SC stimulation induced fEPSPs. Microglia activation induced a significant upscaling of fEPSP amplitude and slope in the CA1 region. Two‐way repeated measures ANOVA, *n =* 9–12 recorded hemisphere brain slices per group, *p < .05

**FIGURE 7:**
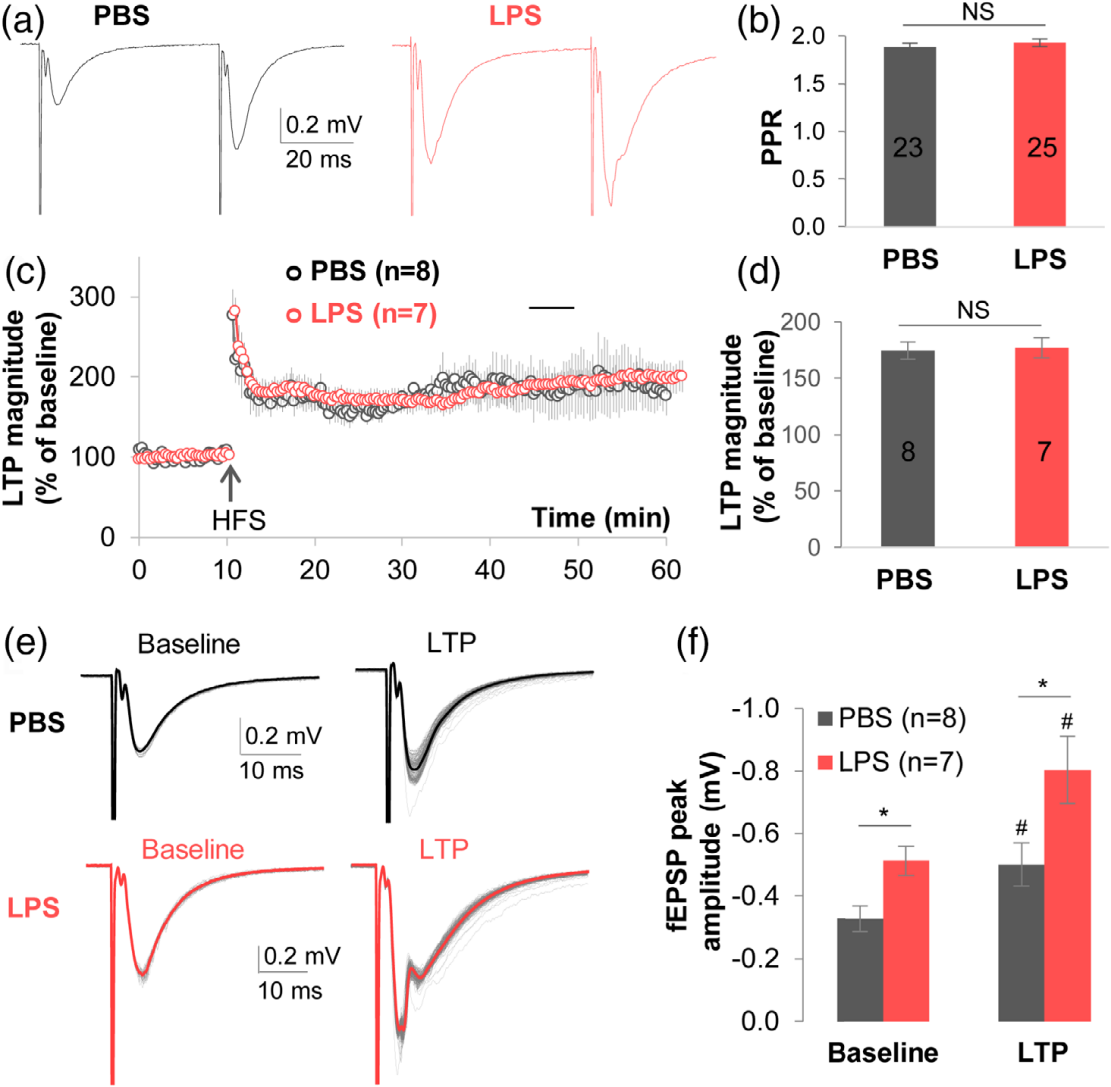
Microglia priming with LPS elevates synaptic transmission without affecting synaptic plasticity. (a) Representative traces of paired‐pulse facilitation (PPF) experiments induced in CA1 synapses in each group. Two consecutive SC stimulations at 40 ms interval were applied to SC to determine the ratio of PPF. The fEPSP amplitudes were markedly higher in LPS injected mice than in PBS controls. (b) The paired‐pulse ratio (PPR) did not differ between LPS and PBS groups. (c) Prior to high frequency stimulation (HFS), 5 min of baseline data was established from stably recorded fEPSP slope. After two trains of 100 Hz HFS (arrow), LTP was induced in both groups in the CA1 region. The fEPSPs were continuously recorded for 50–60 min after HFS, and the potentiation of fEPSP slope from 36 to 40 min post HFS were compared to baseline and between the two groups. (d) Quantification showing the percentage of induced LTP magnitude did not differ between PBS and LPS groups in (c). (e) Representative overlaid recordings of fEPSPs in the baseline, that is, 0–10 min before HFS in (c), and LTP after HFS, that is, 11–55 min in (c), in PBS and LPS groups. (f) Microglia activation by LPS significantly up‐scaled CA3 to CA1 synaptic transmission at both baseline and during LTP recording, #*p* < .05 comparing LTP to baseline in the same group, paired‐sample Student's *t*‐test; *p < .05 comparing between groups, two‐sided Student's *t*‐test. The numbers in the graphs indicate how many recorded hemisphere brain slices in each group

**FIGURE 8:**
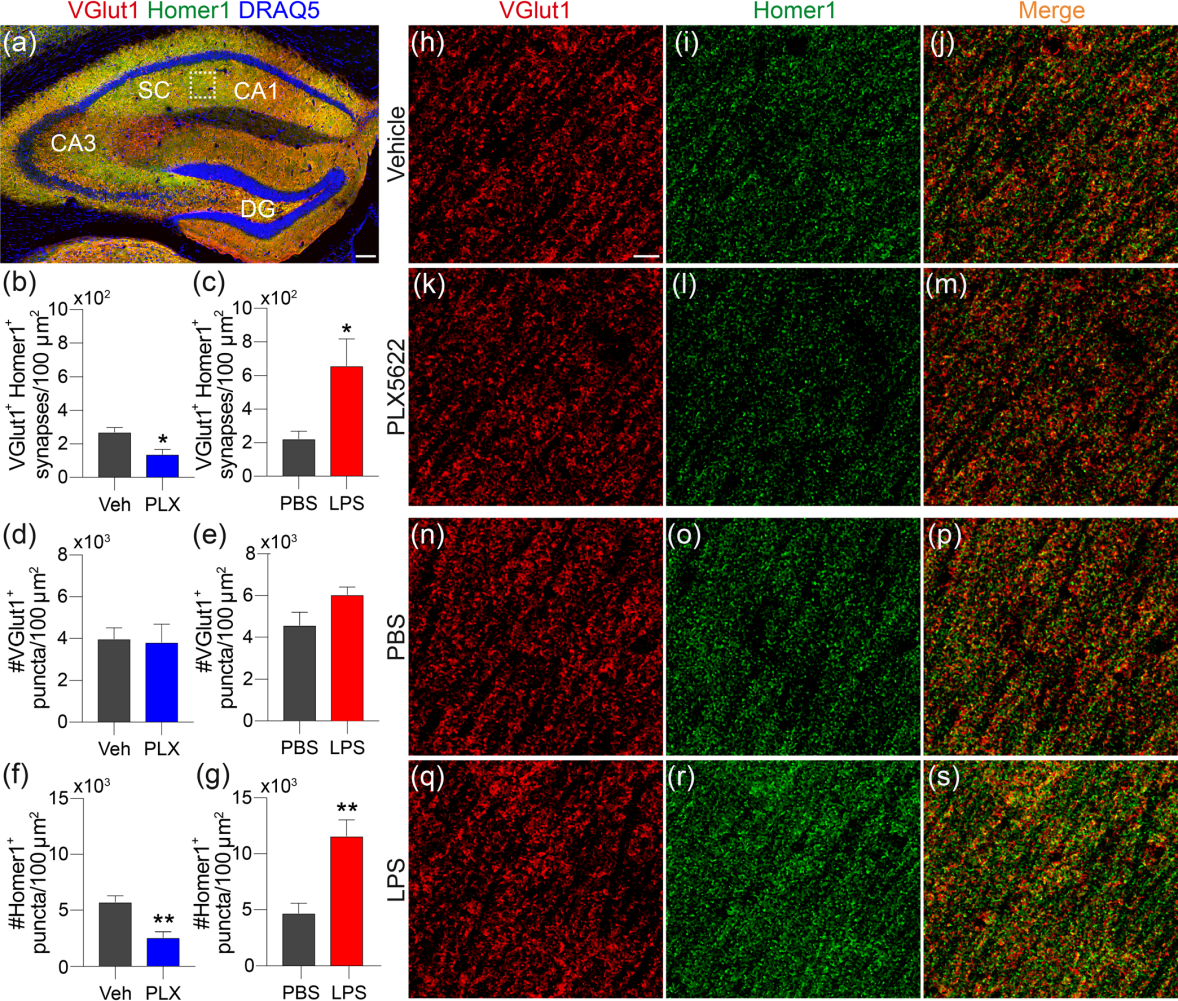
Microglia depletion reduces, whereas microglia activation increases, excitatory synapses and Homer1 expression in the stratum radiatum of the hippocampus. (a) Low magnification image of the hippocampus (vehicle control mouse) stained for Vglut1, Homer1 and DRAQ5. The boxed region indicates the area analyzed for synaptic densities. Scale bar = 75 μm. (b,c) Quantification of synaptic densities revealed microglia depletion reduces (b) whereas microglia activation increases (c) synaptic densities. (d,e) Neither microglia depletion (d) nor activation (e) affected Vglut1^+^ puncta number. (f,g) Microglia depletion reduced (f) but microglia activation increased (g) the number of Homer1^+^ puncta in the stratum radiatum. (h–s) Representative high magnification images of Vglut1 (h, k, n, q), Homer1 (i, l, o, r) and merged images (j, p, s) for mice in each group. Scale bar = 5 μm. (b–g) Two‐sided Student's *t*‐tests, *n =* 4 mice/group, *p < .05, **p < .01

To activate brain microglia without causing neurotoxicity (Chen et al., 2012; Freria et al., 2020), mice were randomized to receive either systemic lipopolysaccharide (LPS, *E. coli*, serotype 055:B5, Sigma, 1 mg/kg i.p.) for four consecutive days, or vehicle injections (0.1 M phosphate buffered saline, PBS). To confirm LPS bioactivity, body weights were recorded daily before injections and before euthanasia on the fifth day (Seemann et al., 2017) (Figures 5, 6, 7, 8 and S4).

### 2.2 Electrophysiology

#### 2.2.1 Hippocampal slice preparation

Hippocampal slices were prepared as described previously (Zhong et al., 2016). Briefly, after anesthesia with 8% chloral hydrate in 0.9% NaCl, mouse brains were rapidly removed from skulls and placed into ice‐cold oxygenated (95% O_2_/5% CO_2_) slice cutting aCSF with reduced Ca^2+^ and increased Mg^2+^ (in mM): 125 NaCl, 3.5 KCl, 25 NaHCO_3_, 1.25 NaH_2_PO_4_, 0.1 CaCl_2_, 3 MgCl_2_, and 10 Glucose. Coronal hippocampal slices (250 μm thickness for astrocyte recording and 400 μm for field potential recording) were cut at 4°C with a Vibratome (Ted Pella Microslicer Zero 1 N) and transferred to oxygenated standard aCSF (in mM): 125 NaCl, 25 NaHCO_3_, 1.25 NaH_2_PO_4_, 3.5 KCl, 2 CaCl_2_, 1 MgCl_2_, and 10 Glucose, osmolality, 295 ± 5 mOsm; pH 7.3–7.4) Slices were recovered at room temperature (RT) for at least 1 h for astrocyte recording, or recovered at 34°C for 1 h and then kept in RT before neuronal field potential recording.

#### 2.2.2 SR101 staining

For identification of live astrocytes in situ, recovered hippocampal slices were transferred to a slice‐holding basket in a beaker containing 0.6 μΜ sulforhodamine 101 (SR101) in aCSF, and incubated at 34°C for 30 min as described previously (Du et al., 2015; Du et al., 2018). The slices were transferred back to normal aCSF at RT before the experiment.

#### 2.2.3 Astrocyte recording

A hippocampal slice was transferred to the recording chamber (RC‐22, Warner Instruments, Holliston, MA) mounted on an Olympus BX51WI microscope with constant perfusion of oxygenated aCSF (2.0 ml/min). A fluorescent imaging system, Polychrome V system (Till Photonics, Germany) was used for high‐resolution identification of SR101^+^ astrocytes. A multiClamp 700B amplifier operated by pClamp 9.2 software (Molecular Devices, Sunnyvale, CA) was used, digitized at 2–10 kHz, and filtered at 2 kHz for whole‐cell patch clamp recording. Borosilicate glass pipettes (outer diameter: 1.5 mm, Warner Instrument, Hamden, CT) were pulled from a Micropipette Puller (Model P‐97, Sutter Instrument Company). The recording electrodes had a resistance of 2–5 MΩ when filled with the electrode solution containing (in mM) 140 K‐gluconate, 13.4 Na‐gluconate, 0.5 CaCl_2_, 1.0 MgCl_2_, 5 EGTA, 10 HEPES, 3 Mg‐ATP, and 0.3 Na‐GTP (280 ± 5 mOsm, pH 7.25–7.27) (Kelly et al., 2009; Ma et al., 2012). A minimum of 2 GΩ seal resistance was required before rupturing the membrane for whole‐cell configuration. The liquid junction potential was compensated for prior to forming the cell‐attached mode for all recordings. The *V*_M_ was recorded under current‐clamp mode for 5 min. The current profile was recorded under voltage clamp mode using 20 mV voltage steps from –180 to +20 mV for 25 ms recording duration. The rectification index (RI) was calculated by dividing the current amplitudes induced by +20 mV (*I*_1_) over ‐ 180 mV (*I*_2_) (Figures S3 and S5). We have previously demonstrated that the “Membrane test” protocol is unable to compute the membrane resistance (*R*_M_) and the access resistance (*R*_a_) from the total membrane input resistance (*R*_in_) in low *R*_M_ astrocytes (Ma et al., 2014). Thus, we monitored *R*_in_ using the “Resistance test” protocol available in PClamp 9.2 software (pulse: ‐ 63 pA/600 ms). Recordings were discarded if the initial *R*_in_ was greater than 20 MΩ or varied greater than 10% during recording.

To record astrocyte syncytial isopotentiality, a Na^+^‐based electrode solution ([Na^+^]_P_) was made by equimolar substitution of K‐gluconate with Na‐gluconate. The pH was adjusted to 7.25–7.27 with NaOH. The recorded cell was ruptured under current clamp mode after reaching 2 GΩ seal. The *V*_M, initial_ stands for the *V*_M_ recorded right after breaking‐in of the cell membrane as cell's resting *V*_M_. The *V*_M, steady‐state_ stands for the steady‐state *V*_M_ 10 ~ 15 min afterward where the Na^+^ substitution with intracellular K^+^ reaches equilibrium, which is a readout of the syncytial coupling strength (*S*) based on our published computational modeling (Kiyoshi et al., 2018; Ma et al., 2016). Recordings were discarded if the initial *R*_in_ was greater than 50 MΩ or varied greater than 10% during recording.

#### 2.2.4 Dye coupling assay

Tracer loading was used to analyze dye coupling in the astrocyte network (Figure 2). The K^+^‐based electrode solution containing a gap junction permeable tracer (biocytin, 0.5 mg/ml) was used to patch astrocytes in the central part of the stratum radiatum in CA1 hippocampus. After whole‐cell mode was formed, astrocytes were recorded in current‐clamp mode for 20 min to allow biocytin dialysis. The *R*_in_ was monitored using the “Resistance test” every 5 min during the recording. Recorded slices were fixed with 4% paraformaldehyde for 1 h at room temperature. To assure efficient and equivalent tracer dialysis between recorded cells and patch electrode, slices were discarded if the initial *R*_in_ was greater than 50 MΩ or varied greater than 10% during recording.

To visualize the biocytin‐filled cells in recorded slices, fixed slices were processed at room temperature as described previously (Eitelmann et al., 2020; Xu et al., 2010). Briefly, slices were washed with PBS, incubated 0.25% triton X‐100 for 30 min, washed again in PBS, incubated with 1:1200 Cy2‐streptavidin (Jackson Immuno Research Labs) in PBS for 4 h, followed by washing in PBS. Slices were incubated in Hoechst 33342 (2 μg/ml, Thermo Scientific) for nuclei staining. After a final wash in PBS, slices were put on glass slides and then coverslipped with Immumount media (Thermo Fisher Scientific).

#### 2.2.5 Extracellular field potential recording

A hippocampal slice was placed in a temperature‐controlled chamber mounted on an Olympus BX51WI microscope equipped with infrared differential interference (IR‐DIC) and were perfused with oxygenated aCSF (2.0 ml/min). The temperature of the perfusing aCSF was controlled at 28–32°C by the Automatic Temperature Controller (TC‐324B, Warner Instruments) during recording. Extracellular field excitatory postsynaptic potentials (fEPSPs) were recorded in stratum radiatum of the CA1 area. Stimuli, 0.1 ms pulse width at 0.05 Hz, were delivered to the Schaffer collateral (SC) axons in the CA1 region through a tungsten concentric bipolar electrode. Recording electrodes were filled with aCSF plus 100 μM picrotoxin (PTX) with a final resistance of 1–2 MΩ. Graded electrical stimulations from 0 to 1.0 mA with 0.1 mA increment were delivered to SC, and the evoked fEPSPs were recorded at 200 ~ 300 μm away from the stimulating electrode. Used the stimulation intensity at the level where ~30% of the maximal amplitude of fEPSPs was induced to establish a basal level of fEPSPs (baseline). fEPSPs were recorded for at least 10 min to acquire a stable baseline. An averaged slope of the fEPSPs, that is, from the range of 20%–80%, over a 5‐min duration was established as a baseline and considered to be 100%. LTP was induced by tetanic stimulation of SC, that is, two consecutive trains at 100 Hz for 1 s that was separated by 20 s. The following slope changes in response to LTP stimulus were expressed as a percentage of this baseline (normalized slopes). An averaged slope of fEPSPs evoked between 36 and 40 min after high frequency stimulation was used for statistical comparisons. Recordings with variations higher than 20% of baseline fEPSPs before LTP stimulus were discarded. The paired‐pulse facilitation (PPF) was performed by the delivery of two identical intensity stimuli at an interval of 40 ms. The ratio of paired‐pulse facilitation (PPR) was calculated using the slope values of the two fEPSPs: the slope of the second fEPSP divided by the slope of the first fEPSP.

### 2.3 Immunohistochemistry

#### 2.3.1 Tissue preparation

In recorded mice, the rostral and caudal portion of the brain, and adjacent hippocampal sections to recorded section, were fixed in 4% paraformaldehyde (PFA) for a minimum of 24 h. Additional littermate mice were perfused with 0.1 M PBS for 1 min followed by 4% PFA for 6 min and 24 h post‐fixation in PFA. All samples were then washed in 0.1 M PBS before incubation in 30% sucrose for 48 h. Samples were embedded in Tissue‐Tek optimal cutting temperature medium (VWR International), and rapidly frozen on dry ice. Brain tissue sections were cut along the coronal axis in series (10 μm thick, 10 slides per series) using a Microm cryostat (HM 505 E) and collected onto SuperFrost Plus slides (Thermo Fisher Scientific), except for connexin‐labeled tissue (Figure 3), which was cut at 30 μm thickness and collected as floating sections into a 96 well plate in 0.1 M PBS.

#### 2.3.2 Fluorescence immunolabeling

Slides were dried at RT for 2 h, rinsed in 0.1 M PBS (3 × 4 min) then blocked for 1 h at RT with 0.1 M PBS containing 4% BSA and 0.3% Triton X‐100 (BP^3+^). Sections were incubated overnight at RT with primary antibodies (Table 1) diluted in 0.1 M PBS containing 4% BSA and 0.1% Triton X‐100 (BP^+^). The next day, slides were washed in 0.1 M PBS (3 × 4 min) and incubated with secondary antibodies (Table 1) and DRAQ5 nuclear dye (1:4000, Abcam, ab108410) diluted in BP^+^. After a final round of washing (0.1 M PBS, 3 × 4 min), slides were then coverslipped with Immumount media (Thermo Fisher Scientific).

**TABLE 1:** Immuno‐labeling reagents

Antigen	Host, dilution	RRID	Vendor, catalog number
*Primary antibodies*			
Connexin 30	Rabbit, 1:250	AB_2533979	Thermo Fisher Scientific 71‐2200
Connexin 43	Rabbit, 1:250	AB_2533973	Thermo Fisher Scientific 71‐0700
GFAP	Rabbit, 1:500	AB_10013382	Dako, Z0334
Iba‐1	Rabbit, 1:500	AB_839504	Wako, 019‐19741
P2RY12	Rabbit, 1:1000	AB_2298886	Anaspec, AS‐55043A
Vglut1	Guinea pig, 1:500	AB_887878	Synaptic Systems,135 304
Homer1	Rabbit, 1:500	AB_887730	Synaptic Systems #160 003
*Secondary amplification*			
Rabbit IgG‐488	Goat, 1:500	AB_2576217	Thermo Fisher Scientific, A‐11034
Rabbit IgG‐546	Goat, 1:500	AB_2534093	Thermo Fisher Scientific, A‐11035
Guinea pig IgG‐568	Goat, 1:500	AB_141954	Thermo Fisher Scientific, A‐11075
Rabbit IgG‐Biotin	Goat, 1:500	AB_228337	Thermo Fisher Scientific, 31,822

#### 2.3.3 Immunoperoxidase labeling

Slides were dried at RT and rinsed in 0.1 M PBS, then incubated at RT in methanol containing 6% H_2_O_2_ for 30 min to quench endogenous peroxidase activity. After washing in 0.1 M PBS (3 × 4 min), blocking and primary antibody incubation steps were performed as above. Sections were then incubated with biotinylated secondary antibodies for 1 h at RT (Table 1). Bound antibody was visualized using Elite‐ABC reagent (Vector laboratories) with ImmPACT diaminobenzidine as a substrate (Vector Laboratories Cat #SK‐4105). Sections were dehydrated through sequential 2 min incubations in 70%, 70%, 90%, and 100% ethanol solutions, followed by 3 × 2 min incubations in Histoclear. Slides were coverslipped with Permount (Thermo Fisher Scientific).

### 2.4 Imaging and image analysis

#### 2.4.1 Dye coupling assay

Hippocampal slices from the gap junction coupling assay were imaged using a Leica TCS SP8 confocal microscope with 20x magnification. Biocytin and Hoechst nuclear dye were used to identify the target region of interest for imaging. Images were composed of 50 step Z‐stacks of 0.5 μm increments spanning 25 μm. Imaging parameters were kept consistent to minimize intensity variability. Z‐stacks were compressed to create one maximum intensity projection (MIP) for counting biocytin‐Cy2^+^ cells in LAS X software (Lecia Microsystems) and measure of biocytin‐Cy2^+^ cell defined area in ImageJ.

#### 2.4.2 Microglia and astrocytes

The Allen Mouse Brain atlas and DRAQ5^+^ channel were used to locate the motor cortex, sensory cortex, corpus callosum, caudoputamen, lateral septal nucleus, and hippocampal regions in fluorescent specimens labeled with Iba1, P2RY12, or GFAP. Fluorescent samples were imaged using a Leica TCS SP8 confocal microscope with 20× magnification. Two images per brain region were acquired per mouse, each composed of 5‐step Z‐stacks of 2 μm increments spanning 10 μm. Bright‐field images of P2RY12 staining were captured at 10–40× magnification using a Zeiss Axioplan 2 Imaging microscope. Imaging parameters were kept consistent to minimize intensity variability. Z‐stacks were compressed to create one maximum intensity projection (MIP). The MIP image was converted to 8‐bit format in ImageJ. The area of Iba1^+^ or GFAP^+^ staining in each region of interest was selected using the threshold tool in ImageJ and expressed as a percentage of the region area. The “analyze particles” feature in ImageJ was used to count the number of glia in each image, with size limit of 50–2000 μm^2^. For astrocyte cell volume analysis, Z‐stacks were exported to Imaris v9.1 (Bitplane Scientific Software). Surface features were created for the GFAP and DRAQ5 channels then added together to generate three‐dimensional profiles. The DRAQ5 channel was made semi‐transparent. Cell volume statistics for all cells in the stratum radiatum of each animal were averaged to produce an “average cell volume” (in μm^3^) value per mouse.

#### 2.4.3 Gap junction proteins

Fluorescent samples labeled with GFAP, DRAQ5 nuclear dye, and connexin 30 (Cx30) or connexin 43 (Cx43) were imaged using a Leica TCS SP8 confocal microscope with 40× magnification. Images were composed of 20 step Z‐stacks of 1 μm increments spanning 20 μm. Imaging parameters were kept consistent to minimize intensity variability. Z‐stacks were compressed to create one MIP. A 70 μm × 70 μm region of interest was centered on a GFAP^+^ cell with astrocyte morphology. After converting to 8 bit format, Cx30^+^ (or Cx43^+^) puncta within the ROI was selected using the threshold tool. The analyze particles feature was used to count the number of Cx30^+^ or Cx43^+^ puncta in each ROI and expressed as puncta/mm^2^. Data were averaged from 5 ROIs per mouse. For quantifying GFAP^+^ colocalization with Cx30 or Cx43, fluorescent Z‐stack images were exported Imaris v9.1 (Bitplane Scientific Software). The overlap of red (connexin) and green (GFAP) channels on a GFAP^+^ cell with astrocyte morphology was calculated using the colocalization tool and expressed as % colocalization per cell. Data were averaged from 5 astrocytes per mouse.

#### 2.4.4 Synaptic proteins

Fluorescent samples labeled with VGlut2, Homer1, and DRAQ5 nuclear dye were imaged using a Leica TCS SP8 confocal microscope with 40× magnification and 2× optical zoom (80× final magnification). Two images per CA1 region were acquired per mouse, each composed of 30‐step Z‐stacks of 0.33 μm increments spanning 10 μm. Tiling with automatic stitching was used to generate high‐resolution Z‐stacks covering large brain regions. Imaging parameters were kept consistent to minimize intensity variability. Z‐stack steps were compressed serially into groups of three (i.e., steps 1–3, 4–6, etc.) to create 10 MIPs. The “Puncta Analyzer” plugin in ImageJ (Ippolito & Eroglu, 2010) was used to threshold VGlut1^+^ and Homer1^+^ staining and quantify the number of colocalized puncta (i.e., an excitatory synapse) and the overall number of VGlut1^+^ and Homer1^+^ puncta. Data for each mouse is the average of two images per mouse.

### 2.5 Statistical analysis

Electrophysiological data were analyzed using Clampfit 9.0 (Molecular Devices, Sunnyvale, CA) and Origin 8.0 (OriginLab, Northampton, MA) (Figures 1, 2, 4, 5, 6, 7, Figures S3 and S5). After evaluating data for normality and homoscedasticity, two‐sided Student's *t*‐tests were used to compare differences in one variable between two groups (Figures 2, 3, 8 and Figures [Link], [Link]). Two‐Way repeated measures ANOVA was used to compare differences of fiber volley amplitude, fEPSP amplitude and slope in the input–output curves between two groups (Figures 4, 6 and Figures [Link], [Link]). Paired‐sample Student's *t*‐tests were used to compare the baseline fEPSP amplitude/slope and after the high frequency stimulation within the same group in LTP experiments (Figures 5 and 7). The Aspin‐Welch Unequal‐Variance test was used when the variances of the two populations are unequal (Figures 1 and 2). The Mann–Whitney nonparametric test was chosen when data were not normally distributed. For electrophysiological studies, *n =* 4–6 mice were used per group, and the exact number of cells or hippocampal slices recorded from is reported in each figure legend. Immunohistochemical data were analyzed using GraphPad Prism 8 (GraphPad software). Because robust effects of PLX5622 and LPS on brain microglia have been previously characterized (Chen et al., 2012; Dagher et al., 2015), we used *n* = 4 mice/group for anatomical analysis to confirm that young adult mice replicated phenotypes observed in adult mice. Data were analyzed by unpaired Student's two‐tailed *t* test, one‐ or two‐way ANOVA with Bonferroni's post hoc test as appropriate. All data are reported as mean and SEM. Statistical significance was determined at *p* < .05.

## 3 RESULTS

### 3.1 PLX5622 depletes brain microglia

To assess hippocampal electrophysiology in the absence of microglia, we pharmacologically depleted microglia using chow laced with a CSF1R antagonist, PLX5622. Most studies using PLX5622 have been performed in the adult mouse (>P56) (Elmore et al., 2014; Freria et al., 2020; Spangenberg et al., 2019; Willis et al., 2020), which eat more chow per day than younger mice (Ellacott et al., 2010). However, recently published data indicate that similar depletion efficacy can be achieved in younger mice (Basilico et al., 2022). Here, we independently confirm that effective microglia depletion can be achieved in P25‐P45 mouse brains (Figure S1a,b). Specifically, in animals fed Vehicle diet, ramified Iba1^+^ microglia tiled throughout cortical and subcortical brain regions (Figure S1c–j). Consistent with previous reports (Askew et al., 2017; Elmore et al., 2014; Li et al., 2014), two‐dimensional analyses revealed that microglia occupied 4.3% ± 0.2% of the brain at a density of 278 ± 23 cells/mm^2^ (Figure S1k,l). In contrast, in young adult mice fed PLX5622 diet, nearly all (>99%; *p* < .0001 vs. Vehicle diet) microglia were eliminated throughout the brain (Figure S1c–j). Remaining microglia occupied only 0.04 ± 0.009% the brain, or 4 ± 1 cells/mm^2^ (Fig. 1SK, L). To confirm that depleted Iba1^+^ parenchymal cells were indeed resident parenchymal microglia and not monocyte‐derived cells, we stained tissue using the microglia‐specific marker, P2RY12. Consistent with Iba1 data, PLX5622 eliminated all P2RY12^+^ microglia throughout the brain (Figure S2). DRAQ5 nuclear staining indicated that microglia elimination did not disrupt normal brain cytoarchitecture (Figure S1c–j), confirming previous observations in adult and young adult mice (Dagher et al., 2015; Elmore et al., 2015).

### 3.2 Microglia ablation does not alter astrocyte K^+^ channel function

Having confirmed that PLX5622 eliminates microglia in young adult mice, we tested the effect of microglia elimination on hippocampal electrophysiology. Since several studies have already proved that microglia are important in regulating synaptic transmission in the hippocampus, primarily through effects on dendritic spines and/or neuronal soma, we explored the effects of microglia on a novel cellular determinant of neuronal excitability and synaptic transmission in the hippocampus – the astrocyte.

Guided by SR101 staining, viable hippocampal astrocytes were easily identified in situ, enabling recordings from individual astrocytes (Nimmerjahn et al., 2004) (Figure S3). Altered astrocyte resting membrane potential (*V*_M_) can be used as a proxy for changes in basal K^+^ conductance (Dallerac et al., 2013). Microglia elimination had no effect on astrocyte *V*_M_ (Vehicle *V*_M_ = −78.2 ± 0.25 mV; *n =* 21 astrocytes vs. PLX5622 *V*_M_ = −77.5 ± 0.36 mV; *n =* 26 astrocytes, *p* = .349, Figure S3b). Although there are various K^+^ channels with diverse activation kinetics, a key characteristic of astrocyte K^+^ conductance is a linear current‐to‐voltage (*I*‐*V*) relationship (Du et al., 2015). Therefore, we used voltage‐clamp to examine the current–voltage profile of individual hippocampal astrocytes. Microglia ablation did not alter the linear *I*‐*V* relationship (Figure S3c,d). To confirm this observation, we compared the rectification index direction (RI) between the two groups by calculating the outward: inward current ratio. A linear *I*‐*V* relationship corresponds to a RI value close to 1, indicating nearly equal absolute values of outward and inward current under equivalent command of voltage of opposite polarity. Functionally, this means an equivalent capacity of the astrocyte membrane to mediate K^+^ influx and efflux (Zhou et al., 2021). Changes in certain K^+^ channel subtypes shift the RI value away from 1 (Du et al., 2016). There was no difference in the RI values of astrocytes between groups (Vehicle RI = 1.0 ± 0.02 (*n =* 11) vs. PLX5622 RI = 1.0 ± 0.01 (*n =* 15), *p* = .437; Figure S3e). Taken together, these data suggest that microglia do not affect homeostatic astrocyte K^+^ channel expression or conductance.

### 3.3 Microglia ablation weakens astrocyte gap junctional coupling

A crucial mechanism for maintaining extracellular K^+^ homeostasis is astrocyte syncytial isopotentiality, where astrocytes constantly equalize their *V*_M_ through gap junctional coupling (Ma et al., 2016). Syncytial isopotentiality can be experimentally detected by K^+^‐free/Na^+^ based electrode solution ([Na^+^]_P_), and the [Na^+^]_P_ recorded *V*_M_ can be correlated to astrocyte gap junctional coupling strength using a computational model (Kiyoshi et al., 2018; Ma et al., 2016; Stephan et al., 2021) (Figure 1a,d). Although microglia ablation does not alter astrocyte K^+^ channel function under basal conditions (Figure S3), we explored whether microglia participate in maintaining astrocyte gap junctional coupling and syncytial isopotentiality under conditions that mimic activation of the astrocyte syncytium (i.e., using Na^+^ challenge). It should be noted that a change in syncytial isopotentiality is the most sensitive and only reliable readout of the functional state of astrocyte syncytial coupling (Du et al., 2020; Stephan et al., 2021). Indeed, the membrane of mature astrocytes is intrinsically “leaky” due to abundant expression of leak type K^+^ channels. As such, dual patch recordings result in ~80% error during measurements making this latter technique valid only for demonstrating the existence and not the strength of astrocyte coupling (Ma et al., 2014; Zhou et al., 2009). The intrinsic “leakiness” of astrocyte membranes also means that the input impedance and I‐V relationship of astrocytes is mostly established by the intrinsic properties of membrane ion channels, not the gap junctions located at the distal end of astrocyte processes. So, input impedance is also not a reliable readout of astrocyte syncytial coupling (Du et al., 2015; Stephan et al., 2021).

We used K^+^‐free/Na^+^ based electrode solution ([Na^+^]_P_) to challenge individual astrocytes with an intracellular influx of Na^+^, then recorded the strength of gap junctional coupling in the astrocyte syncytium (Du et al., 2020; Ma et al., 2016). In these experiments, the initial *V*_M_ (*V*_M, initial_) value was read instantly after rupturing the membrane of recorded astrocytes, which reflects the intact state of resting *V*_M_ of the recorded cell. In line with data in Figure S3, *V*_M, initial_ values before [Na^+^]_P_ challenge were not different between groups (vehicle = −78.5 ± 0.38 mV, (*n =* 15) vs. PLX5622 = −77.4 ± 0.49 mV, (*n =* 15), *p* = .764) (Figure 1b,c).

During Na^+^ challenge, a functional syncytium compensates for the Na^+^ challenge‐induced loss of *V*_M, initial_ by re‐establishing a quasi‐physiological *V*_M_ that is then maintained at a steady‐state level (*V*_M, steady‐state_) (Figure 1a). Conversely, impairment of syncytial isopotentiality manifests as a partial or total loss of *V*_M, steady‐state_ that drifts upward (becoming more depolarized) (Kiyoshi et al., 2018; Stephan et al., 2021). Recorded astrocytes from mice with microglia (i.e., fed Vehicle) maintained their *V*_M, steady‐state_, indicating a functional syncytium with strong electrical coupling between astrocytes. However, without microglia (fed PLX5622 diet), the *V*_M, steady‐state_ of astrocytes during [Na^+^]_P_ challenge shifted significantly toward depolarization, indicating a disrupted astrocyte syncytium (Vehicle *V*_M, steady‐state_ = −72.4 ± 0.44 mV, (*n =* 15) vs. PLX5622 *V*_M, steady‐state_ = −67.7 ± 1.11 mV, (*n =* 14), *p* = .001) (Figure 1b,c). Indeed, according to the *V*_M, steady‐state_ ‐ coupling strength relationship, without microglia, astrocyte gap junctional coupling strength (*S*) was reduced 77.5% (Figure 1d) These data reveal that microglia control gap junctional coupling between astrocytes and are crucial for maintenance of astrocyte syncytial isopotentiality.

If astrocyte syncytial isopotentiality is disrupted by microglia depletion, then structural indices of astrocyte network connectivity also are expected to be impaired. To test this hypothesis, we performed a dye coupling assay. In this experiment, target astrocytes were patched with an electrode solution containing biocytin and visualized using Cy2‐streptavidin. This allows astrocytes within a network that are connected to the patched astrocyte to be easily visualized then counted. In control slices, patched astrocytes were densely surrounded by biocytin‐Cy2^+^ astrocytes with frequent biocytin‐Cy2^+^ astrocytes located distant to the patched cell (Figure 2a,a'). However, without microglia, large areas devoid of biocytin‐Cy2^+^ labeling (yellow dashed lines) were evident adjacent to patched astrocytes and remote (satellite) biocytin‐Cy2^+^ cells were rare (Figure 2b,b'). Quantification confirmed that the number of biocytin‐coupled biocytin‐Cy2^+^ cells was significantly reduced (~3‐fold reduction) without microglia (vehicle: 34 ± 4.0 vs. PLX5622 10 ± 1.6 cells/slice, *p* = .00005) (Figure 2a–c). We also noted that astrocyte processes immediately adjacent to the patched astrocyte appeared better resolved when microglia were depleted (Figure 2a,b). Since the amount of biocytin loading was equal across assays (i.e., a 20 min whole‐cell dialysis), the better‐resolved astrocyte processes appeared to be a result of more biocytin accumulation within fewer astrocytes in the decoupled syncytia devoid of microglia. Quantification confirmed that the total biocytin‐Cy2^+^ area (inside white dashed lines in Figure 2a,b) was reduced without microglia (Vehicle 0.075 ± 0.0048 vs. PLX5622 0.039 ± 0.0054 mm^2^, *p* = .00004) (Figure 2a,b,d), indicating less spreading of dye to distant neighbors and greater dye retention in close neighbors.

For an individual astrocyte, 7–9 directly coupled neighbors are required to maintain the cell in an isopotential network (Ma et al., 2016), the loss of this requisite explains an impaired syncytial coupling from *V*_M_, _steady‐state_ measurement (Figure 1b,c). However, depleting microglia did not affect the relative density (*p* = .30) or volume (*p* = .82) of GFAP^+^ astrocytes in the mouse hippocampus (Figure 2e–h), indicating unaltered astrocyte spatial organization and cell morphology. Thus, the reduced network connectivity of astrocytes following microglia is due to impaired cell–cell communication and not disruption of cell tiling or an increase in astrocyte cell death or atrophy.

### 3.4 Microglia influence gap junctional coupling between astrocytes by regulating expression of astrocyte connexins

To understand how loss of microglia influences the structure and function of the hippocampal astrocyte network (Figures 1 and 2), we analyzed the expression of major gap junction proteins surrounding and contacting GFAP^+^ astrocytes in the hippocampus stratum radiatum. Without microglia, 45% fewer Cx30^+^ puncta decorated regions around astrocytes (vehicle = 8152 ± 1209 vs. 4491 ± 644.5 puncta/mm^2^, *p* = .016) (Figure 3a–g). Since microglia can also express connexins (Gajardo‐Gomez et al., 2016), which could account for reduced field expression, we assessed whether astrocyte‐specific gap junction expression was also reduced. Three‐dimensional image analysis of GFAP^+^ astrocytes colocalized with Cx30^+^ puncta revealed that without microglia, astrocyte‐specific expression of Cx30 was reduced 53% (vehicle = 1.213 ± 0.194 vs. 0.5647 ± 0.1896% colocalization, *p* = .0422) (Figure 3a–h).

To determine whether other key gap junction proteins were affected, we also measured Cx43^+^ gap junctions. Similar to Cx30, without microglia, the number of Cx43^+^ puncta was reduced 35% (vehicle = 7108 ± 534.4 vs. 4516 ± 404.8 puncta/mm^2^, *p* = .0028) (Figure 3i–o), and astrocyte‐specific expression of Cx43 was reduced 63% (vehicle = 0.9431 0.1915 vs. .3545 0.05063% colocalization, *p* = .0061) (Figure 3i–p). Together, these data indicate that microglia regulate astrocyte gap junctional coupling and connexin expression.

### 3.5 Microglia depletion down‐scales synaptic transmission from CA3 to CA1

Data in Figures 1, 2, 3 show that microglia are essential for maintaining astrocyte syncytial isopotentiality and gap junctional coupling. Because normal neuronal function requires intact astrocyte coupling, we extended our investigation to evaluate the effects of microglia depletion on synaptic transmission in the hippocampus.

Excitatory postsynaptic potential (EPSP), resulting from flow of Na^+^/Ca^2+^ into post‐synaptic AMPA/NMDA receptors, determines the likelihood of generating axonal action potentials for signal relay. We recorded extracellular field EPSPs (fEPSPs) in response to electrical stimulation in the stratum radiatum of CA1 (Figure 4a,b). The fiber volley amplitudes triggered by stimulation were comparable between Vehicle and PLX5622 groups across increasing stimulus intensities (*p* > .05, Figure 4c), indicating that microglia ablation does not alter the integrity of glutamatergic axons or the amount of activated pre‐synaptic fibers projecting from CA3 onto CA1 synapses. However, microglia ablation significantly reduced fEPSPs. Indeed, although in both groups fEPSP amplitude increased with increasing stimulus intensity until a plateau was reached (beyond 0.5 mA), mice fed PLX5622 could not generate fEPSP amplitudes comparable to mice fed Vehicle (e.g., fEPSP amplitude at 0.5 mA of stimulation intensity, Vehicle = −0.94 ± 0.11 mV, (*n =* 7) vs. PLX5622 = −0.58 ± 0.07 mV (*n =* 10), *p* = .014) (Figure 4d). Overall, mice fed PLX5622 showed a ~ 40% reduction in the fEPSP slope at the stimulation intensity of 0.6–1.0 mA (*p* < .05, Figure 4e). Together, these data indicate that microglia are needed for optimal CA3 → CA1 excitatory synaptic transmission.

### 3.6 Microglia ablation weakens the strength of synaptic transmission but does not impair synaptic plasticity

Next, we asked whether the absence of microglia affects hippocampal synaptic plasticity. We first examined the paired‐pulse facilitation (PPF) between CA3‐CA1 synapses. PPF is a form of short‐term plasticity, where after a presynaptic neuron receives two impulses in close succession, the magnitude of the post‐synaptic potential will be larger after the second impulse. The mechanisms underlying PPF are exclusively pre‐synaptic, arising from build‐up of presynaptic Ca^2+^ from the first impulse that triggers a larger amount of vesicular glutamate release during the second impulse (Zucker & Regehr, 2002). The paired‐pulse ratio (PPR) was assessed to evaluate presynaptic release‐related short‐term plasticity. In contrast to recent data (Basilico et al., 2022) (see Discussion), we found that the PPR was unaffected by microglia ablation (vehicle = 1.99 ± 0.05; *n =* 27 vs. PLX5622 = 1.87 ± 0.06; *n =* 28, *p* = .118) (Figure 5a,b). These data indicate that acute ablation of microglia does not impair presynaptic function, at least in terms of activity‐dependent vesicular glutamate release.

We next examined the impact of microglia on long‐term potentiation (LTP) in CA3‐CA1 synapses. LTP is a persistent strengthening of synaptic activity that produces a long‐lasting increase in signal transmission between two neurons. Since others have shown microglia presence can either decrease (Basilico et al., 2022; Corsi et al., 2022; Yegla et al., 2021), or increase (Zhou et al., 2011) neurotransmission (see also, Discussion), we sought to understand the relationship between microglia depletion, astrocyte network disruption, and LTP. We observed that the magnitude of LTP was not different between groups (Vehicle = 175 ± 15.6% vs. PLX5622 = 186 ± 16.0%; *n =* 6/group, *p* = .649; Figure 5c,d), indicating that the induction of LTP does not require microglia. However, microglia ablation reduced the fEPSP peak amplitude, both at baseline and during LTP. Specifically, the peak amplitude of fEPSP at baseline was −0.37 ± 0.031 mV in the Vehicle group versus −0.21 ± 0.020 mV in the PLX5622 group (*p* = .0016), and the peak amplitude of fEPSP during LTP was −0.66 ± 0.059 mV in the vehicle group but −0.45 ± 0.048 mV in the PLX5622 group (*p* = .022) (*n =* 6/group; Figure 5e,f). This is consistent with the overall downscaling of fEPSPs in mice fed PLX5622 diet (Figure 4d,e). Taken together, these data indicate that microglia do not affect short‐term presynaptic plasticity in the form of PPF and LTP, but without microglia, downscaling of fEPSP and reduction of HFS‐induced amplitude of fEPSP emerge in the LTP paradigm. Therefore, microglia are necessary to maintain not only a functional astrocyte network, but also basal levels of glutamatergic synaptic transmission in the hippocampus.

### 3.7 LPS primes microglia in the brain

To further determine the dynamic range and ability of microglia to regulate astrocyte gap junction coupling and synaptic transmission, we tested whether microglia can enhance astrocyte gap junctional coupling, hippocampal synaptic transmission, or synaptic plasticity. To prime brain microglia, young adult mice were injected (i.p.) with four consecutive daily doses of LPS (LPSx4). This protocol causes morphological and functional changes in microglia without eliciting leukocyte recruitment and without causing injury or toxicity to the CNS (Chen et al., 2012; Freria et al., 2020; Haruwaka et al., 2019; Lacroix et al., 1998). Control animals were injected with four consecutive daily doses of PBS i.p (Figure S4). We confirmed the bioactivity of LPS using body weight measurements and immunohistochemistry. The weight of mice injected with PBS steadily increased with time, whereas mice given LPS lost >10% of their baseline body weight on injection days 2–4 (Figure S4a, *p* < .01). Ramified microglia with thin processes were observed throughout brain regions in PBS mice (Figure S4b–h), including the hippocampus (Figure S4n). In contrast, primed microglia with amoeboid shape and thickened processes were observed throughout cortical and subcortical regions of the brain of LPSx4 mice (Figure S4b,c,i–m), including in the hippocampus (Figure S4o). On average, LPSx4 increased the proportional area occupied by microglia 3.7‐fold (PBS = 4.4 ± 0.3% [*n =* 4] vs. LPS = 16.1 ± 0.6% [*n =* 7], *p* < .0001), and increased number of microglia 2.3‐fold (PBS = 279 ± 22 cells/mm^2^ [*n =* 7] vs. LPSx4 = 625 ± 36 cells/mm^2^ [*n =* 4] vs. *p* < .0001).

### 3.8 Microglia priming does not affect astrocyte K^+^ channel function or syncytial isopotentiality

Microglial priming with LPSx4 did not change the resting *V*_M_ of hippocampal astrocytes (PBS = −78.4 ± 0.57 mV [*n =* 7] vs. LPSx4 = −78.7 ± 0.28 mV [*n =* 6], *p* = .615) (Figure S5a). The current profile showed a similar linear *I*‐*V* relationship in astrocytes from both groups (Figure S5b,c). This was further confirmed by comparable RI values in both groups (PBS = 1.0 ± 0.01, *n =* 7 vs. LPSx4 = 1.0 ± 0.02 [*n =* 5], *p* = .243) (Figure S5d). These data, like those obtained after microglia ablation (Figure S3), indicate that microglia do not affect the resting membrane potential of astrocytes. Interestingly, unlike microglia depletion, microglia priming did not change the astrocyte syncytial isopotentiality. Specifically, during the [Na^+^]_P_ challenge, the *V*_M, steady‐state_ was −72.2 ± 0.64 mV in the PBS group (*n =* 10), and −72.5 ± 0.92 mV in the LPSx4 group (*n =* 12; *p* = .815) (Figure S5e,f). Taken together, these results suggest that microglia are required for maintaining basal astrocyte syncytial isopotentiality, but priming microglia does not augment astrocyte syncytial coupling beyond baseline.

### 3.9 Microglia priming up‐scales synaptic transmission in the hippocampus

Although microglia depletion reduced astrocytic coupling and fEPSPs (Figures 4 and 5), microglia priming did not affect astrocyte syncytial isopotentiality (Figure S5). However, priming microglia has been shown to facilitate synaptic strength and excitatory neurotransmission (Chen et al., 2012; Clark et al., 2015; Corsi et al., 2022). Thus, the mechanisms by which activated microglia promote neurotransmission and synaptic scaling (i.e., a coordinated increase in strength of all synaptic inputs onto a neuron) are likely related but distinct from those that regulate baseline astrocyte coupling. Therefore, we next tested whether microglia activation could strengthen synaptic transmission without concomitantly strengthening the astrocyte network.

When we measured synaptic transmission in the presence of LPSx4‐primed microglia, there was no change in fiber volley amplitude data indicating that priming microglia does not affect the functional integrity of CA3 axonal projections to CA1 (Figure 6a,b). However, microglia priming significantly enhanced CA3 → CA1 glutamatergic synaptic transmission. Specifically, in the input–output curve with increasing stimulus intensity, the fEPSP amplitude increased in mice with primed microglia relative to mice with resting (presumed homeostatic) microglia (e.g., fEPSP amplitude at 0.6 mA of stimulation intensity, PBS = −0.98 ± 0.09 mV [*n =* 12] vs. LPSx4 = − 1.36 ± 0.12 mV [*n =* 9], *p* = .026) (Figure 6a,c). Overall, priming microglia with LPSx4 increased the fEPSP slope 40%–50% at the “plateau” stimulation intensities of 0.6–1.0 mA (*p* < .05, Figure 6a,d). These data indicate that microglial priming, associated with a 2.3‐fold increase in microglia, up‐scales CA3 → CA1 synaptic transmission. Together with the data in Figures 4 and 5, our results indicate that bi‐directional scaling of glutamatergic synaptic transmission in the mouse hippocampus can be achieved by manipulating the number and activation state of microglia.

### 3.10 Microglia priming strengthens synaptic transmission

The paired‐pulse ratio (PPR) study was performed to determine if microglia activation would affect the release probability of presynaptic glutamate. Similar to our data showing that microglia depletion does not affect the PPR (Figure 5), priming microglia with LPSx4 did not affect the PPR (PBS = 1.89 ± 0.03 [*n =* 23] vs. LPSx4 = 1.93 ± 0.04 [*n =* 25], *p* = .453) (Figure 7a,b). When these normal PPRs are considered together with data showing that axonal integrity is unaffected by priming microglia (based on normal fiber volley amplitudes; see Figure 7b), we can conclude that microglia regulate synaptic transmission at least in part via post‐synaptic mechanisms (Figure 6b), although presynaptic mechanisms have also been reported (Basilico et al., 2022; Clark et al., 2015).

Subsequent studies show that LTP can be induced in mice, regardless of the presence or activation state of microglia. There was no difference in the percent increase of LTP magnitude over baseline after microglia activation (PBS = 175 ± 7.6% [*n =* 8] vs. LPSx4 = 178 ± 8.9% [*n =* 7], *p* = .832) (Figure 7c,d). However, opposite to the phenotype observed with microglia depletion (Figure 5), the fEPSP peak amplitude increased both at baseline and during LTP in mice with primed microglia. The fEPSP peak amplitude at baseline was −0.33 ± 0.040 mV in the PBS group versus −0.50 ± 0.068 mV in the LPSx4 group (*p* = .048), and the fEPSP peak amplitude during LTP was −0.51 ± 0.046 mV in the PBS group versus −0.80 ± 0.107 mV in the LPSx4 group (*p* = .029) (PBS group *n =* 8, LPS group *n =* 7) (Figure 7e,f). This is consistent with the observed up‐scaling of fEPSPs in mice with primed microglia (Figure 6c,d). Taken together, data in Figures 6 and 7 indicate that when primed or activated, microglia can enhance glutamatergic synaptic transmission in the hippocampus, most likely via modulation of post‐synaptic signaling.

### 3.11 Microglia depletion reduces but microglia priming increases post‐synaptic Homer1

To explain the weakened or strengthened synaptic transmission caused by microglia depletion or activation, respectively, we examined the CA1 stratum radiatum neuropil, where dendritic trees are located, for expression of Vglut1 and Homer1, as readouts for excitatory pre‐ and post‐synaptic markers, respectively. This experiment revealed prominent VGlut1 and Homer1 expression in the CA1 neuropil and surrounding Schaffer collaterals, as described previously (Heise et al., 2016; Li et al., 2020) (Figure 8a). In brains without microglia, the number of excitatory synapses (i.e., VGlut1^+^Homer1^+^ colocalized puncta) was significantly reduced relative to control brain (Vehicle = 270 ± 27 vs. PLX5622 = 138 ± 29 synapses/100 μm^2^, *p* = .016) (Figure 8b,h–m). Conversely, priming microglia with LPSx4 increased the number of excitatory synapses when compared to PBS controls (PBS = 223 ± 45 ± vs. LPS = 659 ± 159 synapses/100 μm^2^, *p* = .039) (Figure 8c,n–s). When pre‐ and post‐synaptic puncta in CA1 were counted independently, neither eliminating microglia (Vehicle = 3976 ± 537 vs. PLX5622 = 3812 ± 870 puncta//100 μm^2^, *p* = .88, Figure 8d) nor priming microglia (PBS = 4572 ± 634 vs. LPSx4 = 6038 ± 368 puncta//100 μm^2^, *p* = .09, Figure 8e) affected pre‐synaptic VGlut1^+^ puncta. Conversely, we observed a marked reduction in post‐synaptic Homer1^+^ puncta in microglia‐depleted mice (Veh = 5737 ± 559 vs. PLX5622 = 2567 ± 498 puncta/100 μm^2^, *p* = .006, Figure 8f), and a robust increase in Homer1^+^ puncta after activating microglia (PBS 4685 ± 905 vs. LPSx4 = 11,585 ± 1437 puncta/100 μm^2^, *p* = .007, Figure 8g). Together, these data support electrophysiological data showing that microglia strengthen synaptic transmission predominantly through a post‐synaptic mechanism, which includes increasing expression of post‐synaptic proteins, that is, Homer1, on dendritic trees in CA1.

## 4 DISCUSSION

Although microglia are known to regulate synapse formation and synaptic plasticity (Araque et al., 2014; Eroglu & Barres, 2010), whether microglia also regulate astrocyte syncytial isopotentiality, and how this might relate to synaptic transmission, is unknown. Here, we show that pharmacological microglia depletion weakens astrocyte syncytial isopotentiality, astrocyte connexin expression, and synaptic transmission but priming microglia can enhance glutamatergic neurotransmission in the hippocampus without affecting astrocyte syncytial isopotentiality. Further, microglia likely scale synaptic transmission through a post‐synaptic mechanism that involves regulation of excitatory post‐synaptic densities.

### 4.1 Microglia are indispensable for operation of the astrocyte network

The extracellular K^+^ concentration ([K^+^]_o_, typically ~3 mM), defines the resting membrane potential of neurons and astrocytes (Somjen, 1979). Neuronal activity triggers a ~ 1 mM local increase in [K^+^]_o_, although hyperactivity (seizures) can increase [K^+^]_o_ up to 12 mM (Bellot‐Saez et al., 2017). Since neuronal activity governs synaptic plasticity and ultimately brain connectivity and function (Schoenenberger et al., 2016), rapid removal of extracellular K^+^ is critical for maintaining brain homeostasis. Major disruptions in K^+^ clearance lead to epilepsy ([K^+^]_o_ > 15 mM) (David et al., 2009), spreading depolarizations, and ischemia‐induced cell death ([K^+^]_o_ 30–80 mM) (Leis et al., 2005). Astrocytes lower [K^+^]_o_ by two major mechanisms: (a) channel‐mediated K^+^ uptake and spatial redistribution through astrocyte networks and (b) active uptake through Na^+^‐K^+^ ATPs (MacAulay, 2020). In the former, physiological expression of K^+^ channels and an intact syncytial isopotentiality are required (Zhou et al., 2021). Manipulating microglia, using either robust in vivo depletion or LPS priming did not alter the functional expression of K^+^ channels or resting membrane potential of individual astrocytes, suggesting that microglia do not influence baseline K^+^ conductance in astrocytes.

However, microglia ablation significantly weakens the ability for astrocytes to equalize their membrane potential in our syncytial isopotentiality measurement (Figure 1). This conclusion was corroborated in a dye‐coupling assay, which revealed >60% decoupling of astrocytes to the syncytia without microglia (Figure 2). The reduced expression of Cx30 and Cx43 without altered astrocyte morphology and spatial organization provides a mechanistic explanation for these functional observations. Microglial modulation of astrocyte connexins has been implied from previous in vitro work showing that treatment of cultured astrocytes with microglia‐conditioned media enhances cellular exchange with the extracellular milieu by increasing Cx43 hemichannel activity; these effects are not observed in Cx43^−/−^ mice or in the presence of hemi‐channel blockers (Gajardo‐Gomez et al., 2016; Retamal et al., 2007b). Although a causal link between impaired astrocyte syncytial coupling, connexin expression, and synaptic transmission was not proved in our studies, all events were direct consequences of microglia depletion, and connexin expression has been shown to directly scale synaptic transmission and plasticity (Pannasch et al., 2011).

Several other mechanisms could also explain how microglia communicate with astrocytes to influence basal levels of syncytial isopotentiality. The astrocyte syncytium optimizes brain glycolysis by regulating levels of glutamate, glutamine, D‐serine, and ATP. Microglia could influence the astrocyte syncytium by fine‐tuning these astrocyte metabolic activities through regulating astrocyte release of glutamate. Microglia release ATP, which binds to astrocyte P2Y1R receptors, and in turn stimulates astrocytes to release glutamate (Pascual et al., 2012; Yang et al., 2019). Glial‐derived glutamate generates postsynaptic currents through metabotropic glutamate (mGluR5) receptors on neurons, and this activity can be subsequently detected by and influence the astrocyte syncytium (Pascual et al., 2012).

Microglia also directly interact with astrocytes through physical contact and intercellular communication using cytokines, chemokines, complement factors, and receptor‐mediated cross‐talk (Jha et al., 2019; Schafer et al., 2012; Shi et al., 2020; Vainchtein et al., 2018). Experimental and RNA sequencing data indicate that microglia can induce diverse astrocyte receptor transcriptomes, including those important for syncytial isopotentiality (Guttenplan et al., 2021; Liddelow et al., 2017; Yun et al., 2018). Future studies should characterize the electrophysiological behavior, alongside the transcriptional profile, of astrocyte phenotypes in the presence or absence of microglia. Ideally, future studies would also take advantage of advanced transgenic mouse lines (e.g., ALDH1L1‐eGFP mice) (Zhong et al., 2016) to ensure that all astrocyte subtypes are equally identified to avoid any potential bias in astrocyte labeling or subtype identification.

### 4.2 Microglia regulate synaptic transmission through a post‐synaptic mechanism

A striking observation in our study was the robust effect that manipulating microglia has on hippocampal synaptic transmission; eliminating microglia down‐scales, whereas priming microglia up‐scales the strength of synaptic transmission. Apart from maintaining astrocyte syncytial isopotentiality, a likely mechanism for microglia‐dependent synaptic strengthening is through interaction with neurons. For example, neurons secrete IL‐33, which signals to microglial IL‐33R to enhance microglial engulfment of the extracellular matrix and stimulate synaptic plasticity (Nguyen et al., 2020). A role for microglia in controlling peri‐neuronal nets, extracellular matrix structures that encapsulate neurons, is increasingly being recognized in neurodegenerative diseases with impaired synaptic transmission (Crapser et al., 2020; Fawcett et al., 2019; Venturino et al., 2021).

Microglia also release cytokines that modify postsynaptic proteins (Li et al., 2012; Wake et al., 2009). Microglia derived IL‐1β stimulates postsynaptic AMPA receptor expression, which maintains excitatory synaptic transmission and excitatory/inhibitory balance. IL‐1β also induces phosphorylation of NMDA receptor (NR2B) to promote Ca^2+^ entry and LTP induction (Vivani et al., 2003). Our data show that microglia bi‐directionally scale synaptic transmission without affecting LTP, suggesting that modulation of post‐synaptic AMPA receptor expression is responsible for the effects that we observed. Further investigation of post‐synaptic machinery is needed to definitively reveal how microglia influence synaptic transmission.

Our electrophysiological and immunohistochemical data indicate that microglia depletion downscales—whereas primed microglia up‐scale—synaptic transmission through a post‐synaptic mechanism, and that this mechanism includes modulation of Homer1 expression. Published data indicate that microglial processes interact directly with Homer1^+^ synapses throughout the inferior colliculus, but with greater frequency in regions specializing in top‐down corticofugal plasticity (Webb & Orton, 2019), suggesting that a similar mechanism could occur in the hippocampus during synapse maintenance and plasticity. This supports the observation that direct injection of LPS into the rat brain causes a 4‐fold increase in Homer1 expression (Cui et al., 2015). Deficiency of Homer1 in mice causes a significant redistribution of AMPAR GluA2 subunits from dendrites to the soma in hippocampal neurons (Rozov et al., 2012). This indicates that the regulation of post‐synaptic AMPA receptor expression might be the downstream target of Homer1 related synaptic transmission scaling in our study.

The Homer family of molecular scaffolds are central to synaptic plasticity, as they cross‐link structural proteins in the post‐synaptic density (e.g., PSD‐95, Shank) with intracellular signaling cascades (Tu et al., 1998; Tu et al., 1999). Following an initial synaptic event, de novo Homer1 synthesis and scaffold remodeling is required for the long‐term maintenance of synaptic strength for learning and memory consolidation (Clifton et al., 2019). Indeed, Homer1a mutant mice have deficits in contextual memory consolidation and retention (Banerjee et al., 2016; Datko et al., 2017; Inoue et al., 2009), whereas specific overexpression of Homer1b/c in the dorsal hippocampus reverses stress‐induced cognitive deficits (Wagner et al., 2013). Clinical and experimental studies have linked single nucleotide polymorphisms in the Homer1 gene with psychiatric disorders including schizophrenia, major depressive disorder, and autism spectrum disorder (reviewed by Clifton et al. (2019)). Given that Homer1 expression has broad clinical implications, it will be important to determine the mechanisms by which microglia affect Homer1, and other components of postsynaptic membranes.

How microglia control the plasticity of synaptic transmission has received increasing research attention, however, major discrepancies remain. While an inhibitory role of the microglial process to synaptic transmission has been supported by several recent studies (Badimon et al., 2020; Umpierre and Wu 2021), a short‐term potentiation of synaptic transmission by activated microglia (Clark et al., 2015), and a synchronized firing through the displacement of GABAergic synapses by microglial processes have also been reported (Chen et al., 2014). Thus, more studies that target the same question from different angles are of merit.

We first examined the role of microglia in synaptic transmission through ablation microglia, an attenuated strength of synaptic transmission in our results is consistent with three recent studies where microglia were also depleted by PLX5622 (or PLX3397) (Basilico et al., 2022; Corsi et al., 2022; Yegla et al., 2021). However, there are also discrepancies in the results between ours and others. For example, microglia ablation in our hands did not induce noticeable astrogliosis, but an enhanced GFAP expression was observed by Basilico et al. (2022). An increase or decrease in PPR ratio was reported after microglia depletion by others (Basilico et al., 2022; Corsi et al., 2022). In our hands, the PPR ratio was not affected in the hippocampal CA1 region after removal of microglia. Additionally, the NMDA‐dependent postsynaptic LTP was not altered by microglia ablation in our study but enhanced in the report by Basilico et al. (2022). Differences in brain regions (e.g., cortex vs. hippocampus), animal ages (young vs. adult vs. aged), sex of animals used, method of microglia depletion/activation, and different types of neuronal circuits examined could help explain inter‐experimental differences described in various reports. These discrepancies need to be resolved through future studies under more comparable experimental conditions.

Although our study exclusively used young mice, we predict that similar effects of microglia depletion or priming may occur in the adult mouse brain, where microglia also promote spine formation (Miyamoto et al., 2016; Parkhurst et al., 2013). However, the adult brain contains an increased number of mature neurons and spines and reduced short‐term plasticity in younger mice (Mostany et al., 2013), suggesting it may be less resilient to disruptions in microglia physiology. Indeed, even partial microglial depletion is associated with impaired hippocampal synaptic and cognitive function in both young (4–6 month) and aged (23–24 month) rats (Yegla et al., 2021).

In conclusion, we show that microglia are essential for maintaining astrocyte syncytial isopotentiality under physiological conditions. Homeostatic and activated microglia also control synaptic scaling in the adult mouse hippocampus. Microglia‐targeted approaches may improve the understanding and development of therapeutics for a myriad of neurological conditions involving insufficient or excessive neuronal activity and synaptic plasticity.

## CONFLICT OF INTEREST

The authors declare no conflicts of interest.

## Supporting information


**Figure S1**PLX5622 diet depletes microglia throughout mouse brain. (a, b) Experimental timeline (a) and brain regions (b) analyzed. C: Low magnification image showing the cortex of mice fed Vehicle or PLX5622 immunostained for Iba1. Scale bar = 50 μm. (d‐j) Representative confocal images of Iba1 staining showing effective microglia depletion by PLX5622 in the motor cortex (d), somatosensory cortex (e), corpus callosum (f), caudopuatmen (g), lateral septal nucleus (h), CA3 (i) and dentate gyrus (j). Scale bar d‐j (in d) = 300 μm. (k‐l) Quantification revealed a signification reduction in the Iba1^+^ proportional area (k) and number (l) of Iba1^+^ cell bodies in each brain region in mice fed PLX5622. Two‐way ANOVA with Bonferroni post hoc tests, *n =* 4 mice/group, ****p < .0001.

**Figure S2** PLX5622 diet depletes microglia throughout the young adult mouse brain. (a) Experimental timeline. (b) Low magnification image showing the cortex of mice fed Vehicle or PLX5622 immunohistochemically stained for P2RY12. Scale bar = 220 μm. (d‐j) Representative high magnification images of P2RY12 staining in mice fed Vehicle (c‐g) or PLX5622 (h‐l). Microglia were efficiently depleted by PLX5622 in the somatosensory cortex (c vs. h), Motor cortex (d vs. l), corpus callosum (e vs. j), caudoputamen (f vs. k) and lateral septal nucleus (g vs. l). Scale bar = 50 μm. (m, n) Low magnification fluorescent images of the mouse hippocampus stained for P2RY12 in Vehicle (m) or PLX5622 fed mice (n). Scale bar = 150 μm.

**Figure S3** Microglia ablation does not alter astrocyte K^+^ channel conductance. (a) An astrocyte in CA1 was identified in situ based on its small size (<10 μm in diameter), irregular soma shape, and SR101^+^ staining. (b) The resting membrane potential (*V*_M_) of hippocampal astrocytes is comparable between mice fed Vehicle and PLX5622. (c) The whole‐cell K^+^ conductance recorded from two representative astrocytes, one from Vehicle group and the other from PLX5622 diet group as indicated. The voltage commands (*V*_COM_) for membrane conductance activation are shown on the left panel: the cells were held at −80 mV at resting, and then stepped up by 20 mV increments and 25 ms duration from −180 mV to +20 mV. (d) Current to voltage (*I*‐*V*) plots of the whole‐cell K^+^ conductance were constructed from the color‐coded dashed lines in c showing that the astrocytes passive membrane conductance was not altered by microglia ablation. (e) The rectification index (RI), the ratio of current amplitudes activated by *V*_COM_ of +20 mV (*I*_1_) over −180 mV (*I*_2_), were comparable between Vehicle and PLX5622 diet groups. Two‐sided Student's t‐test; *n =* 5–7 recorded cells per group; NS, no significant difference.

**Figure S4** Systemic LPS increases microglia reactivity throughout the young adult mouse brain. (a) Mice were given four consecutive daily doses of i.p. PBS or LPS. Weight loss following injections was used to confirm the bioactivity of LPS. Two‐Way ANOVA with Bonferroni post‐hoc, *n =* 5–7 mice/group, **p < .01. B, C: Quantification revealed LPS increased the proportional area of Iba1^+^ staining (b) and the number of Iba1^+^ cells (c) in various brain regions. Two‐Way ANOVA with Bonferroni post‐hoc, *n =* 4 mice/group, ****p < .0001. (d‐o) Representative images of Iba1 and DRAQ5 nuclear staining in mice injected with PBS (d‐h, n) or LPS (i‐m, o) throughout various brain regions (d‐m), including the hippocampus (n, o). Scale bar d‐m (in d) = 50 μm, n‐o = 100 μm.

**Figure S5** Microglia activation does not alter astrocyte K^+^ conductance nor astrocyte gap junctional coupling. (a) The resting *V*_M_ was comparable in astrocytes from mice injected with PBS and LPS. (b) Whole‐cell astrocyte passive K^+^ conductance was not altered in LPS injected mice compared to PBS control. The voltage commands (*V*_COM_) for membrane K^+^ conductance activation was identical to the one used in Figure 3. (c) Current–voltage plots of the whole‐cell K^+^ conductance constructed from the color‐coded dashed lines in B; the astrocyte passive K^+^ conductance was not altered by LPS injection. (d) The rectification index (RI) values were comparable between PBS and LPS groups. (e) Representative *V*_M_ traces during [Na^+^]_P_ challenge from recorded astrocytes in PBS or LPS groups. (f) Data summary showed no difference in either *V*_M_, _initial_ or *V*_M, steady‐state_ between PBS and LPS groups. Two‐sided Student's t‐test. A and D, the numbers inside the bar graphs indicate how many recorded cells in each group; f, *n =* 10–19 recorded cells in each group, from 4 mice per group.

## Data Availability

Data available on request from the authors.
