# Supplementary figures and images for: Microglia maintain the normal structure and function of the hippocampal astrocyte network
Source: Glia. 2022 Apr 8;70(7):1359–79. doi: 10.1002/glia.24179 (PMC9324808; doi:10.1002/glia.24179)

Figure S1

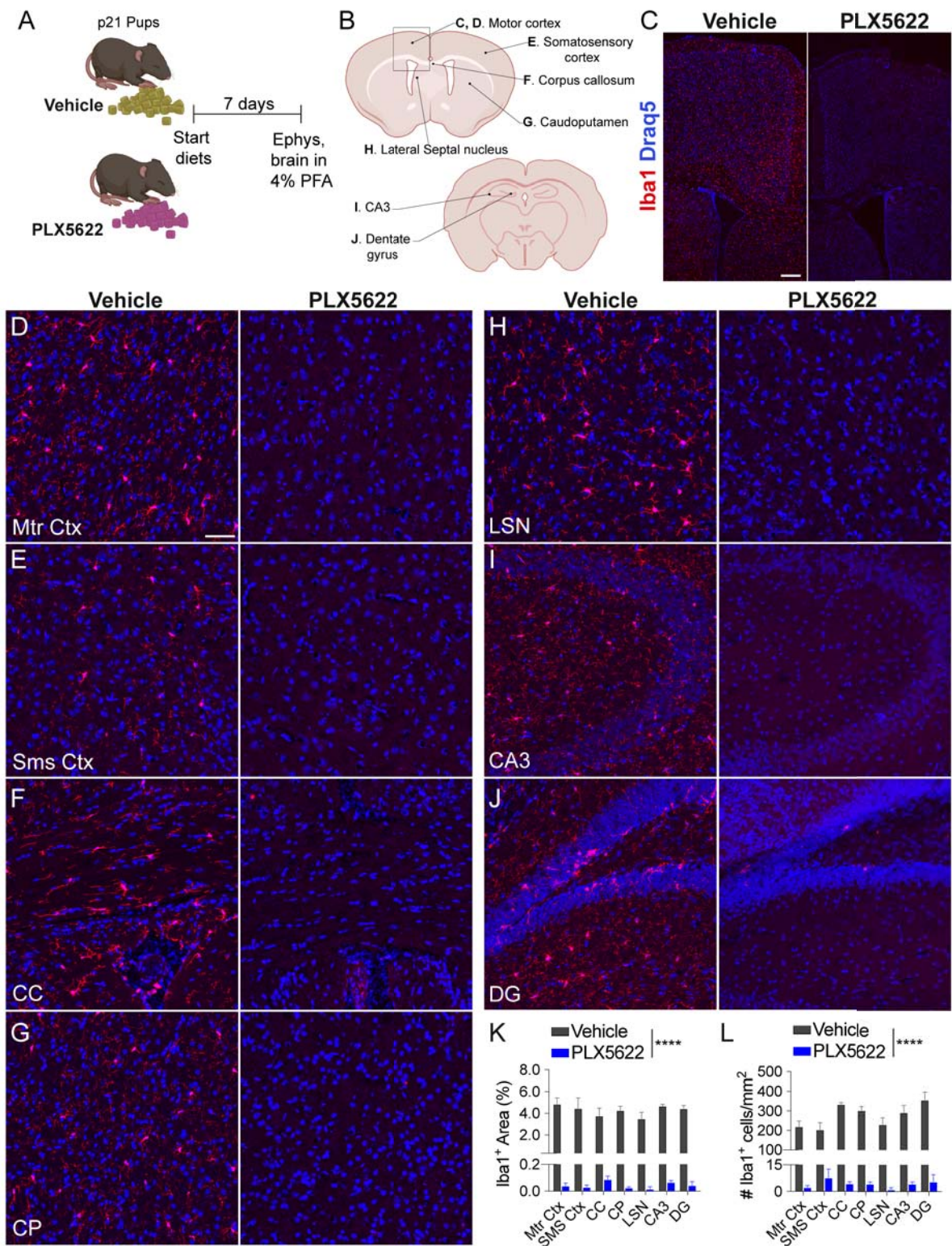

Supplement: Supplementary file 1 — Figure S1PLX5622 diet depletes microglia throughout mouse brain. (a, b) Experimental timeline (a) and brain regions (b) analyzed. C: Low magnification image showing the cortex of mice fed Vehicle or PLX5622 immunostained for Iba1. Scale bar = 50 μm. (d‐j) Representative confocal images of Iba1 staining showing effective microglia depletion by PLX5622 in the motor cortex (d), somatosensory cortex (e), corpus callosum (f), caudopuatmen (g), lateral septal nucleus (h), CA3 (i) and dentate gyrus (j). Scale bar d‐j (in d) = 300 μm. (k‐l) Quantification revealed a signification reduction in the Iba1+ proportional area (k) and number (l) of Iba1+ cell bodies in each brain region in mice fed PLX5622. Two‐way ANOVA with Bonferroni post hoc tests, n = 4 mice/group, ****p < .0001. [file GLIA-70-1359-s004.pdf]

Figure S2

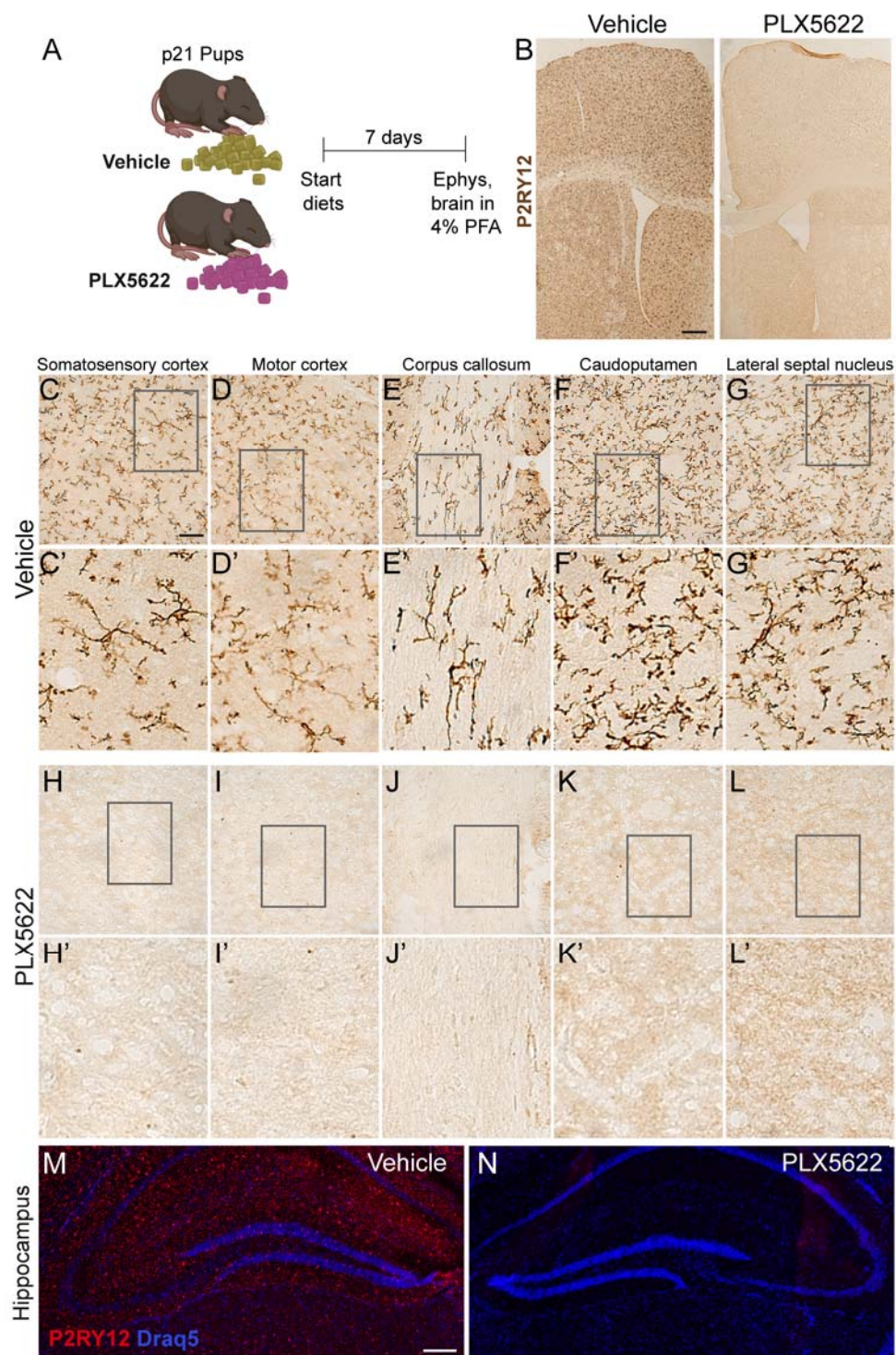

Supplement: Supplementary file 2 — Figure S2 PLX5622 diet depletes microglia throughout the young adult mouse brain. (a) Experimental timeline. (b) Low magnification image showing the cortex of mice fed Vehicle or PLX5622 immunohistochemically stained for P2RY12. Scale bar = 220 μm. (d‐j) Representative high magnification images of P2RY12 staining in mice fed Vehicle (c‐g) or PLX5622 (h‐l). Microglia were efficiently depleted by PLX5622 in the somatosensory cortex (c vs. h), Motor cortex (d vs. l), corpus callosum (e vs. j), caudoputamen (f vs. k) and lateral septal nucleus (g vs. l). Scale bar = 50 μm. (m, n) Low magnification fluorescent images of the mouse hippocampus stained for P2RY12 in Vehicle (m) or PLX5622 fed mice (n). Scale bar = 150 μm. [file GLIA-70-1359-s003.pdf]

# A A SR101<sup>+</sup> astrocyte in CA1 region

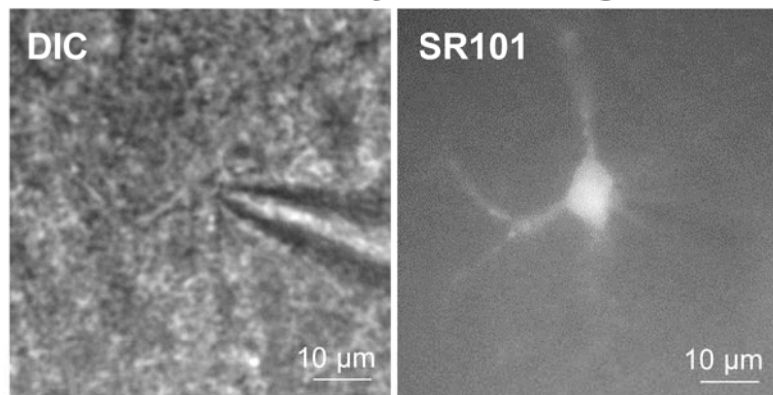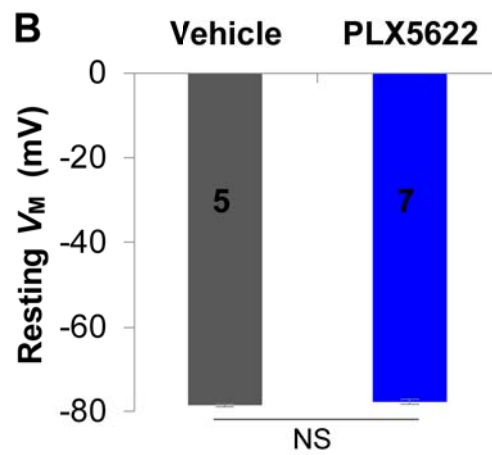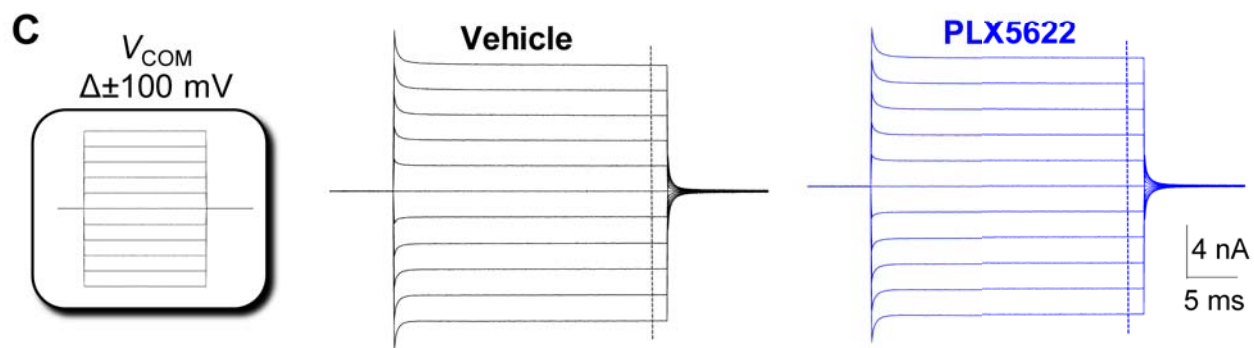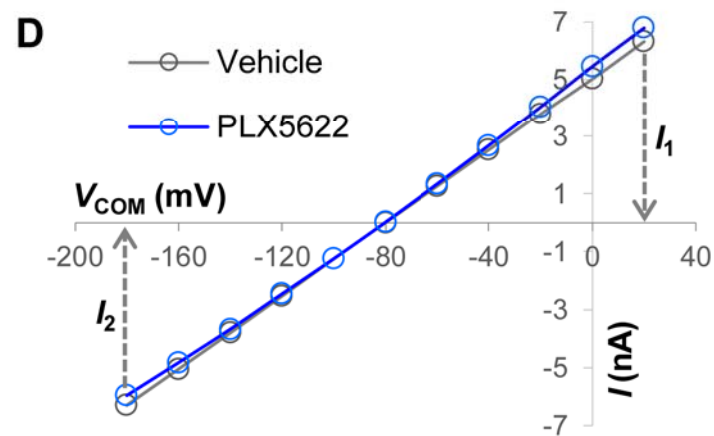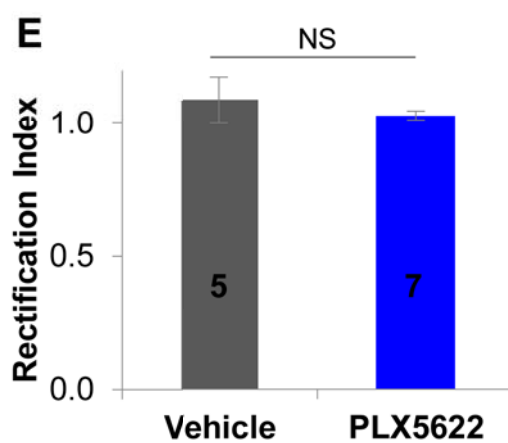

Supplement: Supplementary file 3 — Figure S3 Microglia ablation does not alter astrocyte K+ channel conductance. (a) An astrocyte in CA1 was identified in situ based on its small size (<10 μm in diameter), irregular soma shape, and SR101+ staining. (b) The resting membrane potential (V M) of hippocampal astrocytes is comparable between mice fed Vehicle and PLX5622. (c) The whole‐cell K+ conductance recorded from two representative astrocytes, one from Vehicle group and the other from PLX5622 diet group as indicated. The voltage commands (V COM) for membrane conductance activation are shown on the left panel: the cells were held at −80 mV at resting, and then stepped up by 20 mV increments and 25 ms duration from −180 mV to +20 mV. (d) Current to voltage (I‐V) plots of the whole‐cell K+ conductance were constructed from the color‐coded dashed lines in c showing that the astrocytes passive membrane conductance was not altered by microglia ablation. (e) The rectification index (RI), the ratio of current amplitudes activated by V COM of +20 mV (I 1) over −180 mV (I 2), were comparable between Vehicle and PLX5622 diet groups. Two‐sided Student's t‐test; n = 5–7 recorded cells per group; NS, no significant difference. [file GLIA-70-1359-s001.pdf]

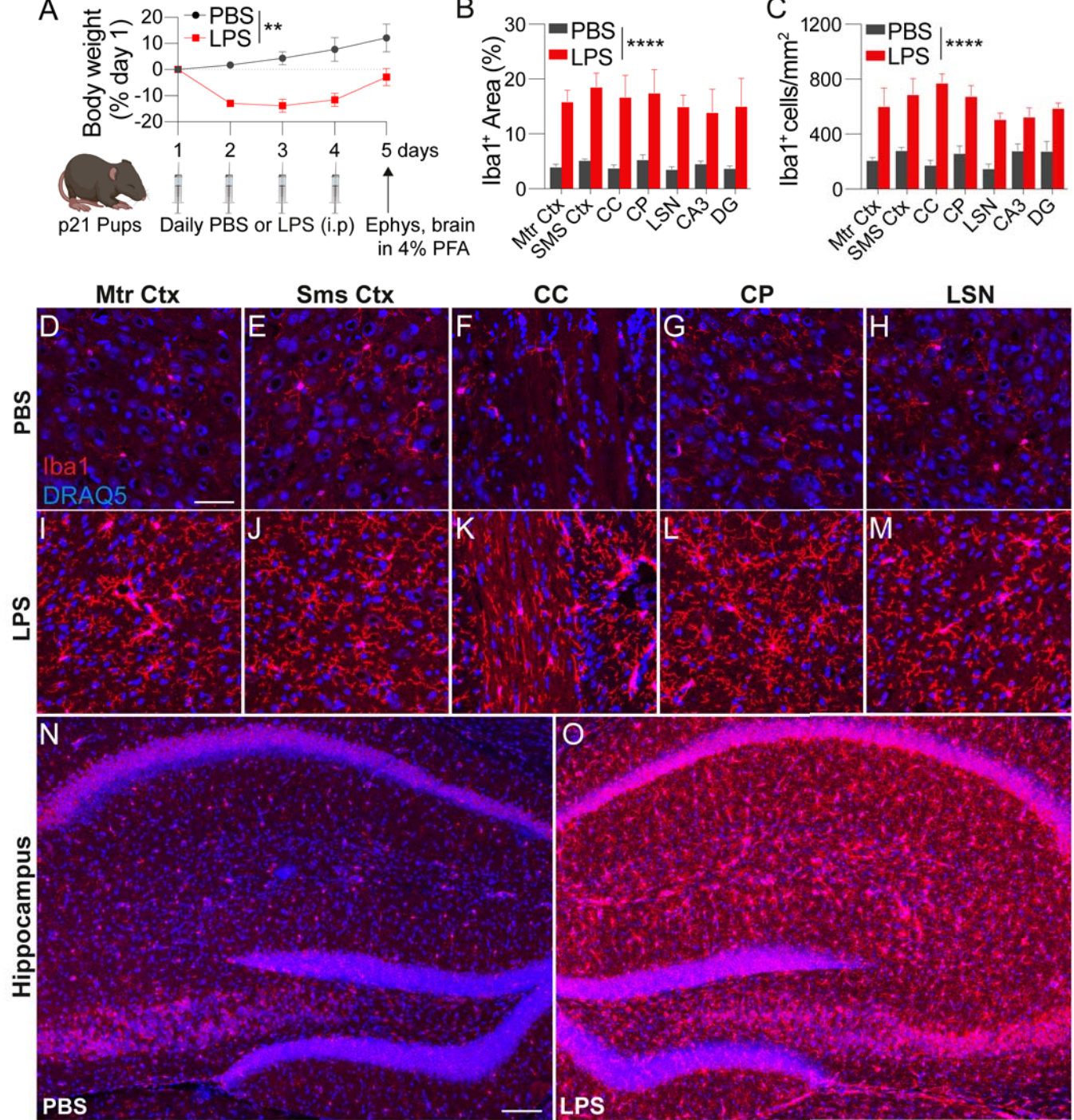

Supplement: Supplementary file 4 — Figure S4 Systemic LPS increases microglia reactivity throughout the young adult mouse brain. (a) Mice were given four consecutive daily doses of i.p. PBS or LPS. Weight loss following injections was used to confirm the bioactivity of LPS. Two‐Way ANOVA with Bonferroni post‐hoc, n = 5–7 mice/group, **p < .01. B, C: Quantification revealed LPS increased the proportional area of Iba1+ staining (b) and the number of Iba1+ cells (c) in various brain regions. Two‐Way ANOVA with Bonferroni post‐hoc, n = 4 mice/group, ****p < .0001. (d‐o) Representative images of Iba1 and DRAQ5 nuclear staining in mice injected with PBS (d‐h, n) or LPS (i‐m, o) throughout various brain regions (d‐m), including the hippocampus (n, o). Scale bar d‐m (in d) = 50 μm, n‐o = 100 μm. [file GLIA-70-1359-s005.pdf]

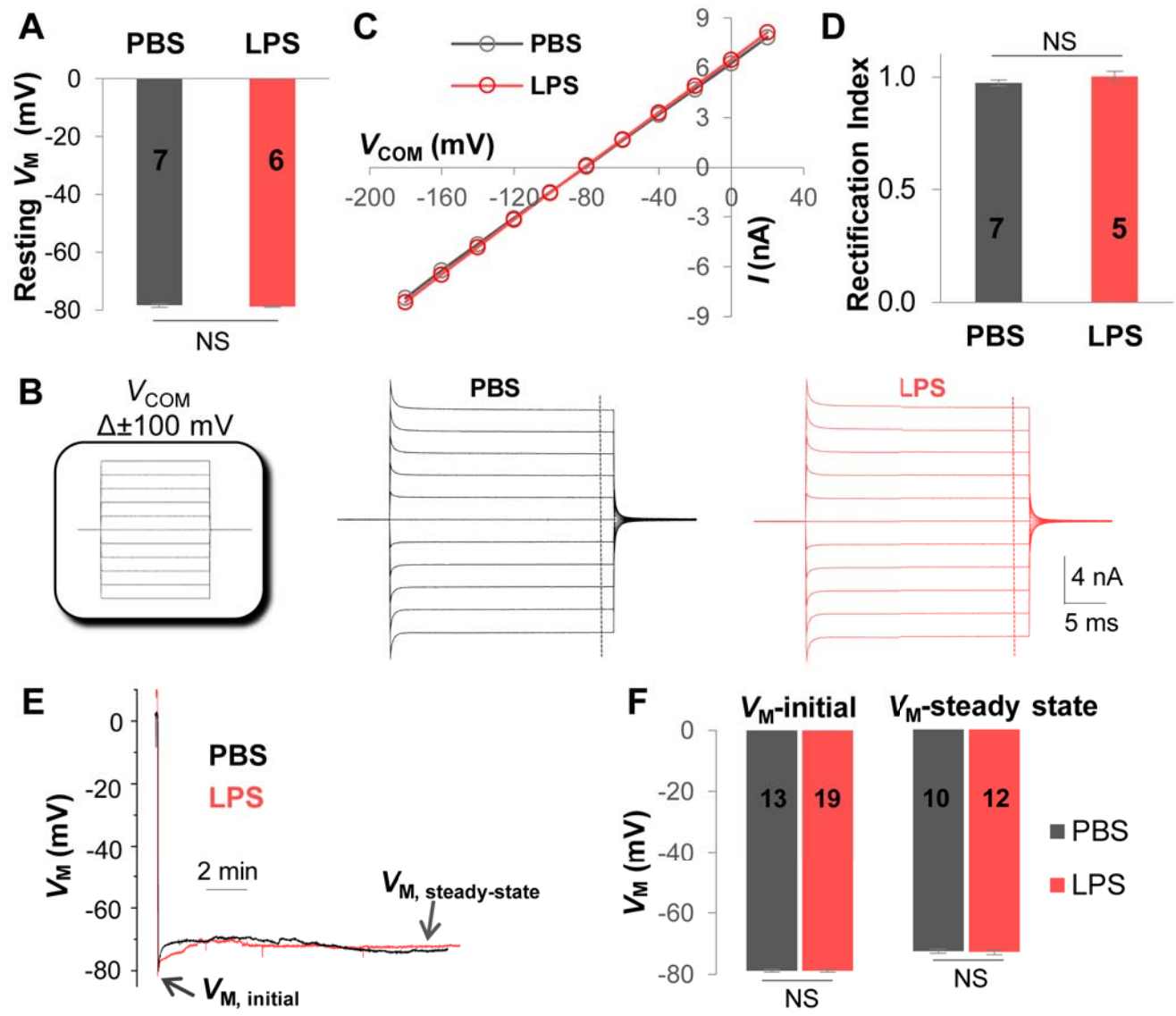

Supplement: Supplementary file 5 — Figure S5 Microglia activation does not alter astrocyte K+ conductance nor astrocyte gap junctional coupling. (a) The resting V M was comparable in astrocytes from mice injected with PBS and LPS. (b) Whole‐cell astrocyte passive K+ conductance was not altered in LPS injected mice compared to PBS control. The voltage commands (V COM) for membrane K+ conductance activation was identical to the one used in Figure 3. (c) Current–voltage plots of the whole‐cell K+ conductance constructed from the color‐coded dashed lines in B; the astrocyte passive K+ conductance was not altered by LPS injection. (d) The rectification index (RI) values were comparable between PBS and LPS groups. (e) Representative V M traces during [Na+]P challenge from recorded astrocytes in PBS or LPS groups. (f) Data summary showed no difference in either V M, initial or V M, steady‐state between PBS and LPS groups. Two‐sided Student's t‐test. A and D, the numbers inside the bar graphs indicate how many recorded cells in each group; f, n = 10–19 recorded cells in each group, from 4 mice per group. [file GLIA-70-1359-s002.pdf]
